# Co‐Designing a Culturally Tailored Nutrition Resource With African Migrant Women and Healthcare Professionals in Australia

**DOI:** 10.1111/hex.70649

**Published:** 2026-03-25

**Authors:** Bolanle R. Olajide, Paige van der Pligt, Vidanka Vasilevski, Fiona H. McKay

**Affiliations:** ^1^ Institute for Health Transformation (IHT), School of Health and Social Development Deakin University Burwood Victoria Australia; ^2^ Department of Allied Health, School of Health Sciences Swinburne University of Technology Hawthorn Victoria Australia; ^3^ School of Health and Social Development Deakin University Burwood Victoria Australia; ^4^ Department of Nutrition and Dietetics Western Health Footscray Victoria Australia; ^5^ School of Nursing & Midwifery, Centre for Quality and Patient Safety Research, Institute for Health Transformation Deakin University Burwood Victoria Australia; ^6^ Western Health St Albans Victoria Australia

**Keywords:** co‐design, cultural tailoring, healthcare providers, migrant women, nutrition resources, pregnancy

## Abstract

**Background:**

Deciding what foods to consume during pregnancy can be overwhelming. Accurate and culturally appropriate nutrition resources are particularly important for migrant women navigating different cultural influences. Research with African migrant women has highlighted the need for tailored pregnancy nutrition resources, yet this need remains unmet. Providing African migrant women with appropriate resources can support informed food choices and contribute to improved maternal and foetal health outcomes. This study explored the views and opinions of African migrant women and healthcare professionals (HCPs) in Australia on the development of a culturally appropriate pregnancy nutrition resource.

**Methods:**

The authors drew on previously prioritised needs for culturally tailored pregnancy nutrition resources as a starting point for co‐design workshops. Four workshops were conducted via Zoom with seven African migrant women and five HCPs. Participants included women and HCPs from an earlier study as well as new participants recruited through convenience sampling. Data were analysed using reflexive thematic analysis, combining deductive and inductive approaches.

**Results:**

Three themes captured the preferred formats and features of a tailored pregnancy nutrition resource. Among the suggested formats, the top four preferred were a mobile application, website, pamphlet, and food guide. Key features included incorporating African foods, identifying suitable alternatives available in Australia, providing information on common cultural food restrictions, and listing where African and substitute foods could be purchased. Across all formats, participants emphasised the importance of concise, visually engaging resources.

**Conclusions:**

Co‐designed, culturally tailored nutrition resources can support women's nutritional needs, foster culturally sensitive care, and improve maternal and foetal health outcomes.

**Patient or Public Contribution:**

African migrant women and HCPs contributed to co‐design workshops, guiding the development of culturally tailored pregnancy nutrition resources.

## 1 Introduction

Australia has long been a destination for migrant populations and is recognised as a desirable country for international migration [1]. In Australia, the African migrant population is growing. Over half a million people of African heritage were living in Australia in 2024, representing 2% of the population [2]. With the growing population is an increasing number of women from African countries who are giving birth in Australia; approximately 3% of all births in 2023 [3]. This changing population highlights the increasing need for antenatal care services that are culturally appropriate and responsive to the unique needs of African migrant communities.

Antenatal care (ANC) is important, enabling women to access essential healthcare during pregnancy. ANC aims to identify and manage conditions that, if left untreated, can result in adverse outcomes [4]. ANC can provide clinical intervention as well as evidence‐based health education, including nutrition information [4]. Tailoring ANC services to meet cultural needs is essential to empower women from diverse backgrounds and improve equity in maternal health. Moreover, pregnancy represents a unique *‘window of opportunity’* for cardiovascular risk stratification and long‐term prevention in women [5].

Receiving culturally tailored nutrition information during ANC is particularly important for migrant women. However, research from the United States of America (USA) and the United Kingdom (UK) has reported that after migration, African migrant women do not always receive nutrition advice that is culturally appropriate or relevant to their needs [6, 7]. A recent study with African migrant women in Australia [8], found that hospital‐provided nutrition materials are not always culturally tailored. A lack of support during pregnancy can contribute to confusion and uncertainty among women, particularly recent migrants, about safe foods that also fulfil cultural needs during pregnancy. This underscores the need for culture‐specific resources that better support women and healthcare professionals (HCPs) during ANC.

Australian studies have demonstrated the effectiveness of co‐designing culturally tailored health resources with migrant and refugee women for a range of healthcare needs including cervical screening education [9] and the creation of multilingual maternal health education videos [10]. Building on this approach, this study sought to use co‐design frameworks to explore suggestions and opinions about a tailored pregnancy nutrition resource with African migrant women and their healthcare providers. Co‐design provides practical and detailed qualitative insights into individuals’ specific knowledge and needs, with a focus on developing solutions [11, 12].

## 2 Methods

This study builds on previous research involving migrant women and HCPs who participated in separate qualitative interviews conducted in 2023 and 2024 [8, 13]. Findings from the previous study highlighted the need for culturally tailored pregnancy nutrition resources. The current study reengaged with the same migrant women and HCP through a series of co‐design workshops to discuss and design a culturally appropriate nutrition resource.

### 2.1 Study Design

This study used a series of workshops, informed by a co‐design method to 1) identify the preferences of women and HCPs for a tailored pregnancy nutrition resource, and 2) explore what constitutes a culturally appropriate and acceptable resource. Co‐design is a participatory approach that allows for the development of interventions that empower users, gives them a voice, and incorporates their cultural needs [14, 15]. Health interventions designed using this method have been found more likely to be culturally appropriate, acceptable, adoptable, and sustainable [16, 17]. This user‐centred method recognises participants as experts of their own experiences and positions them at the centre of the design process [18]. In the current study, women and HCPs were valued as key sources of knowledge who could collaboratively design a new resource [19].

This research adopted an extension of the design thinking framework [20]. The design thinking framework comprises five stages (empathise, define, ideate, prototype, and test), with each stage building on the outputs of preceding stages in an iterative manner, as proposed by Plattner [21]. The design thinking framework allows for the application of agile and problem‐solving techniques. These techniques were used in this research to engage HCPs and women to co‐design potential solutions that address a gap in the provision of culturally appropriate pregnancy nutrition information resources.

**Figure 1:**
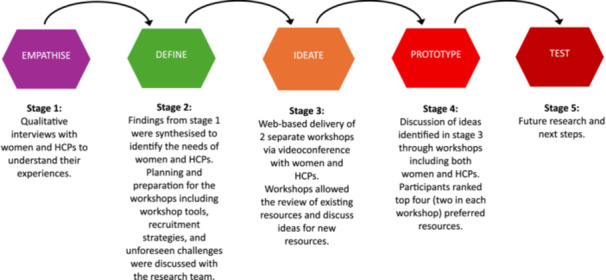
Overview of the co‐design framework and mapping of the study stages (adapted from Plattner [21] and Baxter et al. [22]).

This study employed four of the five stages of this framework (see Figure 1). The *empathise* stage involved understanding the pregnancy experiences and food practices of women and the experiences of HCPs providing pregnancy care to these women. These insights were gathered through previous, separate qualitative interviews [8, 13]. This information was synthesised in the *define* stage to identify the key challenges and needs of both women and HCPs. The findings informed the scope and aims of the co‐design workshops. The *ideation* stage consisted of the co‐design workshops reported here, where women and HCPs participated in separate workshops to identify preferred formats and types of pregnancy nutrition resources. In the *prototype* stage, all ideas generated for culturally tailored pregnancy nutrition resources were compiled and together in a single workshop, participants ranked the preferred resources and reached consensus on the content to be included in the tailored resource.

### 2.2 Setting and Participants

Women were eligible to participate if they were aged 18 years and over, born in Africa and living in Australia, either currently pregnant or had given birth within the past 2 years in Australia, proficient in English, and provided informed consent. English proficiency was self‐assessed via the eligibility survey to enable participants to engage fully in interactive workshops without the use of an interpreter. HCPs were eligible if they had provided antenatal or nutrition care to African migrant pregnant women in Australia.

Participants were primarily recruited from the pool of women and HCPs who had participated in our previous studies and expressed interest in participating in future research (Stage 1 in Figure 1 **)**. To ensure adequate number of the sample, additional participants were recruited.

Participants who were involved in the previous study were sent an invitation email including detail about the workshop, a recruitment flyer, and a link to a Qualtrics survey. The survey contained the Plain Language Statement (PLS), demographic questions, a request to confirm availability and preferred platform (in‐person or online) for the workshop, and a consent form.

Additional participants, who had not participated in our previous studies, were recruited using convenience sampling. A recruitment flyer was shared through the professional and personal networks of the research team. The first author also contacted dietitians across Australia via email to invite them to participate in the workshop, to address a noted gap, as dietitians were not represented among the HCPs in the previous study. Specifically, dietitians who indicated in their profiles that they support women's pregnancy nutrition were contacted. All new participants completed the Qualtrics survey to confirm their eligibility and provide their contact details for workshop participation.

**Figure 2:**
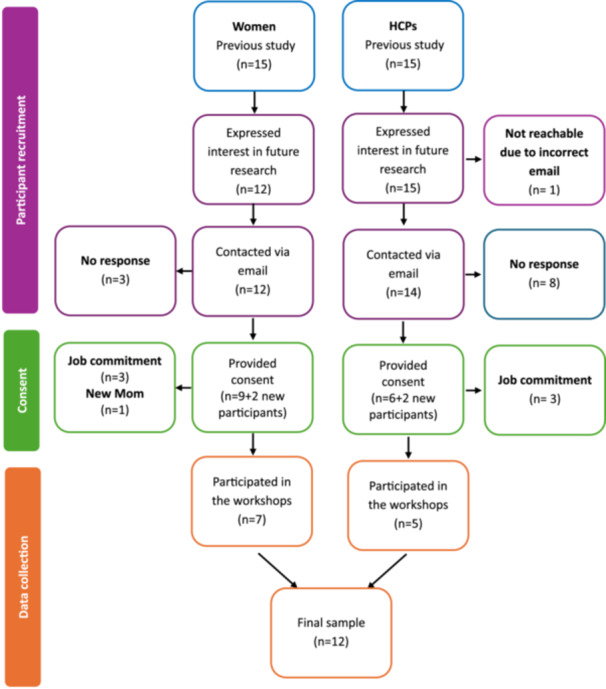
Flow diagram of workshop participants, with reasons for exclusion.

Of the 15 women from the previous study, 9 consented to participate in the current study, and 2 new participants consented and were included. Ultimately, 7 women took part in the workshops (3 were unavailable due to work commitments; 1 had recently given birth). Of the 15 HCPs from the previous study, 6 provided consent and 2 new participants consented and were included. A total of 5 HCPs participated, 3 were unavailable due to work commitments. The participant flow diagram is presented in Figure 2. Twelve participants consisting of seven women and five HCPs participated in the co‐design process. All women had migrated from Nigeria and had been living in Australia for between two and 9 years. Their average age was 34 years (range: 31‐40), and they held either a Bachelor's or a postgraduate degree. The HCP sample included three midwives, an obstetrician, and a dietitian. All HCPs currently worked in a public hospital, with two to eleven years of experience providing care to women. For readability, African migrant women will be referred to as *‘women’ or ‘woman’* throughout this manuscript.

### 2.3 Data Collection

Workshops were conducted between February and May 2025. Ethics approval was obtained from the Deakin University Human Research Ethics Committee (approval number: 2023‐331). A total of 12 participants took part in two of four workshops (2x separate (women's only or HCP only workshops) and 2x joint women and HCP sessions). The anticipated sample size for each group was 4–6 participants, guided by the concept of *‘information power’* [23].

Prior to the initial workshops, both women and HCPs were emailed three existing pregnancy nutrition resources that were commonly available or distributed to women during antenatal care in Australia (see Supporting Information S1: File 1). These included *healthy eating when you're pregnant* [24], *healthy eating during your pregnancy* [25], and *Nutrition + Pregnancy* [26]. The email also included a link to a pre‐workshop video, which provided an overview of what participants could expect during the workshop, including the planned activities.

**Figure 3:**
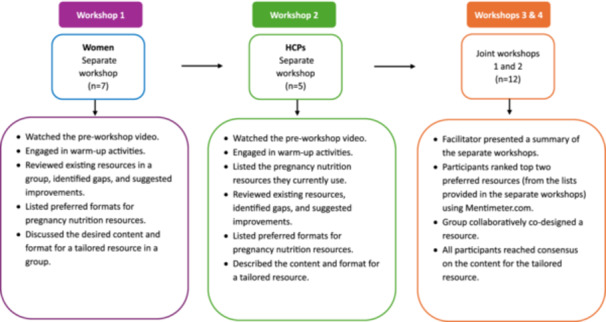
Data collection process across the four workshops.

All participants were given the opportunity to review these resources prior to the workshop, as they formed the basis for the workshop discussions and activities. To facilitate participant preferences, all workshops were conducted via videoconference using the Zoom platform. This approach helped reduce barriers to participation and enabled the researchers to engage with ‘*hard to reach’* populations [27]. A semi‐structured workshop guide was used to conduct the workshops (see Supporting Information S2: File 2). The workshop was facilitated by the first author and two members of the research team. To facilitate participation, the first workshop with women was conducted as a single group session, while the first workshops with HCPs were held individually due to scheduling conflicts. Workshops 3 and 4 were held jointly with both women and HCPs. All 12 participants contributed to the joint workshops. Figure 3 outlines the data collection process across the four workshops.

All workshops were audio‐recorded, lasted between 60 and 115 min, and were transcribed verbatim. As compensation for their time, each participant received a $100 AUD gift card after completing the workshops. The authors also held a series of debriefing sessions before and throughout the data collection process. The trustworthiness of the study was enhanced through member checking. All participants were given the opportunity to review and validate the workshop transcripts [28]. None of the HCPs expressed interest in reviewing their individual workshop transcripts, three participants chose to review the joint workshop transcripts and confirmed its accuracy. None requested any changes.

### 2.4 Data Analysis

Guided by a constructivist paradigm [29, 30], which assumes that reality is socially constructed, workshop data were analysed both deductively and inductively. After the separate workshops (workshop 1 and 2) with women and HCPs, the first author listened to and transcribed verbatim the audio recordings. The transcripts were then manually analysed deductively to identify gaps in the existing pregnancy nutrition resources, suggested areas for improvement, participants’ preferred types of pregnancy nutrition resources, and key elements of a culturally tailored resource.

These initial findings were organised into bullet points and presented to participants in workshop 3 and 4 via a PowerPoint presentation. During workshops 3 and 4, participants validated and agreed that the findings accurately reflected their previous discussions, thereby strengthening the rigour of the analytic process. Following workshops 3 and 4, all transcripts (workshops 1‐4) were coded using NVivo 15 software and analysed inductively using Braun and Clarke's six‐phase approach to reflexive thematic analysis [31]. Separate and joint workshop transcripts were first coded as distinct datasets to preserve the differing perspectives of women and HCPs before being considered together during theme development.

The first author undertook line‐by‐line open coding, generating initial codes close to participants’ words. Codes were then compared within and across datasets, clustered into categories and further to potential themes. Themes were iteratively refined through repeated reading of the transcripts. Although coding was conducted by one author, rigour was enhanced through regular analytic meetings in which the research team reviewed code definitions, challenged interpretations, and collaboratively refined theme boundaries until consensus was reached.

Theme development followed an iterative process of reviewing the themes against the coded extracts and the full dataset, defining the central organising concepts of each theme, and selecting illustrative quotations. The analysis of the separate workshops aimed to explore both groups’ perceptions of existing resources, their preferred resource types, and culturally tailored content. This resulted in the identification of two themes. The analysis of the joint workshop transcripts focused on identifying key features of the collaboratively co‐designed resources and led to one additional theme. A coding tree illustrating the analytical structure is provided in Supporting Information S3: File 3. This collaborative approach through author involvement, participant validation, and iterative refinement enhanced the validity, integrity, and trustworthiness of the overall analysis [32].

## 3 Results

Analysis of the workshop transcripts identified three main themes. The themes include cultural misalignment in existing nutrition resources, translating pregnancy nutrition guidance into culturally relevant resources, and designing practical and usable pregnancy nutrition resources. The overarching finding is that women and HCPs preferred both electronic and paper‐based pregnancy nutrition resources. They also emphasised the importance of including nutrition information specific to African cultures in such a resource. Quotes are used to represent findings and are attributed as follows for clarity: quotes from women will be labelled as *‘woman’* or *‘women’*, while quotes from HCPs will be labelled as *‘HCP’*, followed by the ‘*participant's number’*. Quotes from the joint workshops are attributed to the participants who presented the details of the co‐designed resources on behalf of each small group. This could be either a woman or HCP.

### 3.1 Theme 1: Cultural Misalignment in Existing Nutrition Resources

This theme was derived from discussions conducted in workshops 1 and 2. In both workshops, participants identified gaps in the existing pregnancy nutrition resources and listed their preferences for pregnancy nutrition resources. The most shared concern across both women and HCPs was the lack of culturally relevant information tailored to African women. Women highlighted the absence of guidance on traditional African foods, while HCPs noted the lack of information on food taboos during pregnancy. Women explained that the inclusion of culturally tailored content, especially information on African traditional foods, would increase their willingness to engage with nutrition resources. HCPs expressed that such content would provide practical tools to support women during antenatal care visits.

Women shared that they were generally unfamiliar with the existing pregnancy nutrition resources sent by the facilitators prior to the workshops 1 and 2, although some mentioned having received similar materials during their ANC. Upon reviewing the resources, the women said that they appreciated the structure, particularly the way the materials outlined food groups and provided guidance on appropriate quantities to consume.

‘…we said it is a good one because it gives me a guideline… to know what to do and what not to overdo in order to help you and the baby’ (Woman 1).

Despite these initial positive perceptions, both women and HCPs identified gaps in the existing resources. Both groups highlighted concerns regarding content relevance and appropriateness. The resources were perceived as too general and not aligned with African food practices. Participants noted the absence of information about African foods.

…the kinds of foods that we eat is a bit different from the kinds of foods that are included in these flyers…for me that was the very first culture shock that I had…

(Woman 7)

This view was echoed by an African HCP, who remarked that the listed foods did not reflect what a typical African meal would include.

…they're not speaking directly to what we consider as a meal in Africa.

(HCP 4)

One HCP said that these gaps were also present in other nutrition materials commonly distributed to women in hospital settings.

… the nutritional advice that we have in the materials and the pamphlets that we use are more or less generalised…Without necessarily catering for this particular cohort of African women.

(HCP 2)

While this gap was acknowledged, one HCP noted that although the resources provided to women at their hospital were general, they did include some cultural adaptations.

It's a generic resource that we use for lots of different women from other cultural backgrounds, but we do have some cultural adaptations within it… we have injera and that's the only African specific food that we have in that resource.

(HCP 5)

Women identified the limited range of food suggested to them to prevent illness. For example, women who were iron deficient during pregnancy spoke of the variety of iron rich vegetables available in their home countries, highlighting that the food recommended in pamphlets did not always meet these nutritional needs, or requiring them to consume large quantities to reach the desired nutrient levels.

…there's a lot of leaves that we eat that are very good in iron, which is why you never have that problem back home but here they'll tell you spinach is rich in iron…before you can get that iron that it is rich in, you have to eat like a bucket load of spinach.

(Woman 6)

The HCPs highlighted different problems with the existing resources. For example, they were concerned that the resources were overly detailed. HCPs also pointed out that the food images included in the resources lacked specific descriptions, which could limit their usefulness.

The foods pictures they're not all labelled as to what they are.

(HCP 3)

Participants were asked for their ideas for a new resource or the redesign of existing resources to address identified gaps. Women and HCPs were supportive of the creation of new resources and updating the existing resources.

…modifying this document [pamphlets] by having different links like the way we have different languages like if you're Chinese, you click on this one… adding links so that the information that pertains to the African culture can be found somewhere.

(HCP 4)

One woman saw value in the pregnancy app that had been recommended to her during an antenatal visit. She suggested that such apps could be redesigned to include nutrition information relevant to African women.

…redesigning or including some of those African information in those apps…I used that app very well… the app actually takes me away from Google…

(Woman 4)

Participants were supportive of the development of a newly designed pregnancy nutrition resource. One HCP emphasised the need for a tailored resource that could be implemented consistently across hospitals in Australia.

We need a dedicated external interest group for this to be able to drive resource development that's culturally appropriate, not just for our hospital, but Australia wide.

(HCP 5)

Women were hopeful that the design of new resources would give them a sense of belonging, enhance cultural connection, and provide greater comfort during pregnancy.

…we know that it won't be 100% how we have it back home, but it's also part of the things that will make us feel at home as well because we call Australia home…comfortable for us as possible…

(Woman 2)

Participants mentioned several potential different types of resources that could be developed. These included a mobile phone application, website, pamphlets, posters, pre‐recorded nutrition messages, videos, food guides, meal plans, and recipe books. Both groups identified a preference for pamphlets, posters, and videos. Table 1 provides an overview of the resources suggested, accompanied by quotes explaining the reasons for participants’ preferences.

**Table 1:** List of participants’ preferred resources with reasons for their choices.

Resources	Women	HCPs
Pamphlet	*‘[A] pamphlet that contains pictures for me will be the best because you keep seeing it and it reminds you of what to do…not to do… can have it in your car… on the table in the house…the more you see it, the more you feel eager to use it’*	*‘Easiest for us to have a piece of like an information sheet with the pictures on it, like a pamphlet… that we can go through with the women and then they can take home with them’*
		*‘…a pamphlet feels more collaborative as a practitioner, because it's something that we can look at together and we can circle things and we can add things and we can put sticky notes on it…make it more useful for that person’*
Posters	*‘…when I mean poster, it will be like Australian guide to healthy eating…when you go to doctor's office, you see it on the wall. So the poster will work for doctor's offices’*	*‘When you walk into health facility, we'll have like posters with languages like Chinese. Vietnamese and all of that. So we can have something African in general with regards to the information…’*
Videos	*‘…there was this video package that my hospital gave to me to complete before my delivery. So, we could also incorporate this [cultural nutrition information] into that and should be part of the videos to watch during pregnancy’*	*‘Videos probably having conversations with African women when recording… instead of just recording a video where a dietician or nutritionist is talking about the foods, I think having it in a conversation type… with a pregnant black woman will be great to see…can be shared with other African women’*
		*‘…watch a video from an expert on the topic about some of these things …it may be easier for them to take in. And if we have these resources in different African languages’*
Mobile application	*‘I used what to expect app and it was really useful… if apps like that can carry along resources for our nutrition as well’*	*‘If there was an app that was tailor made for the community, then that would be a great resource for healthcare professionals. And for women as well, to be able to access’*

### 3.2 Theme 2: Translating Pregnancy Nutrition Guidance Into Culturally Relevant Resources

Workshops 1 and 2 explored participants expectations of a culturally relevant pregnancy nutrition resource. Participants described how nutrition information would need to be translated into forms that reflected African foods, practices, and ways of communicating.

Participants described that the resource should include culturally tailored content. Both groups identified similar content to be included, such as African foods available in Australia and/or suitable alternatives, and information about the nutritional benefits of food. Another common suggestion was the inclusion of food quantities in relatable measurements.

Foods that are healthy and that we are familiar with… pictures will work…I think most people can relate to pictures… if there are alternatives that you're familiar with, it won't be too overwhelming.

(Woman 4)

One HCP spoke about including information on the nutritional benefits of alternatives to African foods available in Australia.

…this is an alternative that you can get here in Australia, and you will get the same nutrients…that could help to substitute what they're used to with what is available.

(HCP 2)

Both groups had difference expectations for tailored content. HCPs wanted content that would benefit both themselves and the women they support. For example, they wanted the resource to include information about African food taboos and myths, as well as recommendations of African foods that could healthily be consumed during this period. They emphasised that including such content would raise their awareness and better guide their discussions with women.

…food taboos. As a midwife that was something I had no knowledge of. So if there was something that could go through the most common ones from different areas. And so that we could address them together.

(HCP 1)

One HCP emphasised that a key benefit for women would be the inclusion of information on where to purchase African foods.

…in my experience… recent immigrants who may not be very aware of where to get many of these foods… one of the things that can be considered is information on where to get the foods.

(HCP 2)

Both groups shared similar preferences regarding the language and presentation of the resources. Participants emphasised the need for materials that are concise, visually appealing, and easy to understand. They recommended using plain English, minimising text, and incorporating colours and symbols to enhance relevance and accessibility for both literate and non‐literate women.

…using clear language, clear simple terms and no jargon terms that can easily be understood, like not bulky, eating small frequent meals, not eating foods that have too much fat.

(HCP 4)

One woman expressed a preference for a colourful and attractive resource

… it should be catching like colourful and attractive, so that way, it will make it easy to read.

(Woman 5)

The women described the importance of including content related to physical activity, which was not emphasised by the HCPs.

There was nothing about exercise and from where we come from. We don't really see exercise as a big deal, but also putting that exercise in.

(Woman 4)

### 3.3 Theme 3: Designing Practical and Usable Pregnancy Nutrition Resources

The joint workshops focused on how pregnancy nutrition resources could be designed to be practical and usable for both women and HCPs in everyday settings. During each workshop, participants were asked to rank two resources they would prefer to see developed. Options were based on the suggestions made in workshops 1 and 2. The four highly ranked resources were pamphlets, a mobile application, a website, and a food guide. Participants shared their reasons for ranking these resources. One key reason highlighted by HCPs was the need for tools to facilitate communication with women from African communities.

…we want to do our job the best we can to help support women that walks around or no matter where they come from and having resources and having better insight into what they're actually eating, we need it.

(HCP 5)

Participants highlighted that a mobile application or website were the most accessible formats. The development of a tailored resource in these formats was considered easiest for women to access pregnancy nutrition information whenever needed.

I think an app can be a very, very good resource for women and they can be able to utilise it at any time.

(HCP 4)

**Figure 4:**
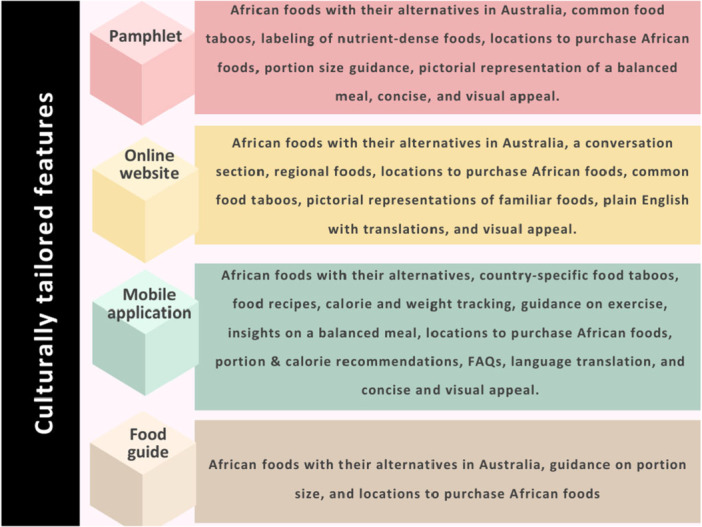
Culturally tailored features of the co‐designed resource types.

Following this initial discussion, participants were divided into small groups to begin the collaborative co‐design of one of the four resources presented. The co‐design of two different resources was begun in each workshop 3 and 4, with a focus on content, design, and language features. Features suggested for each resource type are presented in Figure 4.

In workshop 3, participants discussed the co‐design of a mobile application and a website, proposing features to ensure the tools would be culturally appropriate for women's needs during pregnancy. Suggestions included incorporating African foods from different regions, providing suitable alternatives to common African foods that could be found in Australia, indicating where both African and substitute foods could be purchased, and links to both general and country‐specific nutrition information.

…a section whereby it will be general things that are common to we Africans… when you go into the app. The first thing is going to ask you is what country are you from?…and then you pick your country.

(Woman 5)

They also recommended including information on country‐specific cultural food restrictions and their meanings, noting that these vary widely.

…the taboos will be based on also your country. So while some countries in Africa believe that when you eat eggs…your child will come out bald with no hair. It's not like that in some other countries.

(Woman 5)

They highlighted the importance of addressing misconceptions about pregnancy food taboos.

It would be beneficial if part of the website you have to demystify the common taboos around food in pregnancy. So that people are not avoiding things that are actually nutritious.

(HCP 2)

Additional features suggested for the mobile application included calorie and weight tracking, food recipes with nutritional information, portion and calorie recommendations, guidance on suitable pregnancy exercise, and a section for frequently asked questions (FAQ) related to pregnancy nutrition.

There should be a section for frequently asked questions, there should be a place where you can ask questions and then the FAQ…questions that people have been asking about nutrition in pregnancy.

(Woman 3)

In workshop 4, participants discussed the co‐design of a pamphlet and a food guide. Both sub‐groups suggested including a short list of African foods with suitable alternatives, preparation instructions, and guidance on where to purchase them in Australia.

Traditional foods with good pictures… Western substitute or alternatives for that food… short little recipe list of how to prepare the Western food alternatives so people understand how to use them.

(HCP 1)

The pamphlet sub‐group proposed a simple format with limited information.

…just a basic pamphlet…like an example of a stew, an example of a porridge, an example of a grain…It would have to be really basic. But maybe that's where the app and the website could expand on it as well.

(HCP 1)

They also suggested including information on common food taboos, guidance on appropriate size of meals, identifying nutrient‐dense foods beneficial during pregnancy, and a pictorial representation of a balanced meal using traditional African foods.

we thought the first page should be important nutrients during pregnancy…then a picture of a plate featuring a traditional meal…with a proportion and guide of protein, carbohydrates and fats…what a balanced meal might look like.

(HCP 1)

The food guide sub‐group saw the guide as a tool for outlining appropriate portion sizes and food groups.

Food Guide is going to be a guide, so it should only contain what should be consumed in what proportion and the food classes.

(Woman1)

Presentation and language were also considered important across both workshops. Participants stressed that information should be concise and visually appealing, provided in English, with a translation feature for African languages.

…because most Africans have their own tribal language. But most countries can speak English…It will be fair if it is in English language. Also, maybe if there can be a way to maybe this AI thing that can translate it to your language.

(Woman 5)

Supporting Information S4: File 4 provides an overview of the culturally tailored features accompanied by relevant participant quotes.

## 4 Discussion

High‐quality ANC requires engaging women in decision‐making and recognising them as active participants in optimising their own health [33]. Understanding women's specific needs for pregnancy nutrition information, as well as their preferences regarding how such resources should be developed, is therefore an important first step in promoting adequate and supportive nutrition education. To the best of our knowledge, this study is the first to bring together women and HCPs to co‐design culturally tailored pregnancy nutrition resources in Australia. The participation of both groups in separate and joint workshops enabled the recognition of their respective needs in decision‐making and fostered inclusivity in resource development.

Findings from this study showed that women and HCPs preferred a mix of electronic and paper‐based formats, with participants suggesting a mobile application, website, pamphlet, and food guide as mediums that would best support enhanced nutrition education. Developing any of these resources with the culturally tailored features suggested by participants could help address the challenges African migrant women face in accessing pregnancy nutrition information that meets their needs [6, 7]. Accordingly, any new resource must be both culturally relevant and evidence‐based to ensure it supports healthy dietary choices. Strengthening pregnancy nutrition support may also have benefits beyond immediate maternal and foetal outcomes, extending to women's long‐term cardiometabolic health. Pregnancy is recognised as a critical period in which dietary patterns and metabolic changes can influence future risk of conditions such as obesity and cardiovascular disease [34, 35]. Evidence suggests that suboptimal maternal nutrition is associated with less favourable cardiometabolic profiles later in life, while improved diet quality during pregnancy can support healthier weight trajectories and metabolic function postpartum [36]. For African migrant women, culturally tailored resources that facilitate healthier food choices may therefore contribute not only to safer pregnancies but also to the prevention of chronic disease following childbirth. Framing pregnancy nutrition as an investment in women's lifelong health strengthens the public health relevance of developing culturally responsive interventions.

Incorporating familiar foods is a key strategy for increasing the usability of pregnancy nutrition resources. Participants in this study recommended including African foods with suitable alternatives to common African foods that could be found in Australia. The pregnancy period can be overwhelming for women when navigating what to eat [37]. Providing a tailored resource that features familiar African foods, clear guidance on portion sizes, and nutrient information could help women make confident food decisions. Requests for familiar African foods indicate that nutrition advice is interpreted through cultural identity and everyday food environments rather than biomedical guidance alone [38]. The absence of such foods in existing resources may therefore signal a lack of cultural recognition, which can reduce trust and engagement with services [39, 40]. Similar findings were reported in a study involving migrant women from culturally and linguistically diverse (CALD) backgrounds, where the inclusion of familiar foods in a pregnancy app increased its acceptability [38].

Involving HCPs in the co‐design process in this study, provided valuable suggestions regarding a tailored resource that could be used in a clinical setting. HCPs recommended the inclusion of information on African food taboos and guidance on healthy African foods during pregnancy. Such content could increase HCPs’ awareness of African food practices, supporting cultural understanding and inclusivity in healthcare [39, 40, 41]. Research exploring Swedish midwives’ experiences in providing ANC to migrant women has similarly reported insufficient cultural knowledge to support migrant women effectively [40]. Incorporating African cultural practices in a resource may therefore help strengthen communication, build trust, and encourage preventive health practices, which could contribute to improved outcomes for mothers and babies. These findings highlight a gap between standard nutrition guidelines and the cultural realities encountered in practice, a challenge also reported in studies of midwives working with migrant women [40, 41]. Co‐design appears to function as a mechanism for redistributing expertise between women and HCPs, supporting more person‐centred and culturally safe care.

Presentation and language are important factors for the effectiveness of nutrition resources [42]. Both women and HCPs in this study emphasised the importance of using visuals, concise text, plain English, and translations. Prioritising these elements during resource development may increase accessibility, improve comprehension, and enhance the overall effectiveness of nutrition communication for pregnant women.

### 4.1 Strengths and Limitations

This study is the first to capture women's suggestions and opinions on tailored pregnancy nutrition resources, addressing a gap identified in previous research [6, 7, 43]. A key strength was the inclusion of both women and HCPs in co‐design workshops, which combined clinical expertise with women's experiences to generate rich, in‐depth insights. However, it is important to note that the resources discussed in workshops 3 and 4 were preliminary; participants did not produce a final product, which remains a future step in the development process. The inclusion of only English‐proficient participants may have excluded some African migrant women with limited language proficiency, potentially narrowing cultural representation and perceptions. In addition, the online format may have excluded women with limited digital literacy or financial constraints for the purchase of mobile data, despite efforts to reduce participation barriers through flexible scheduling and remuneration for their time. A further limitation is that all African migrant women were from Nigerian backgrounds; therefore, the preferences for the potential pregnancy nutrition resource may not represent those of other African communities in Australia. Notwithstanding this limitation, the involvement of HCPs with experience caring for women from diverse African backgrounds supported consideration of preferences relevant across a wider African migrant population. The inclusion of HCP of African heritage may have influenced discussions through greater cultural familiarity; however, themes were developed across all participants and refined through team‐based analysis to minimise the influence of individual viewpoints. While the study is situated within the Australian healthcare context, some findings may have relevance to other settings that support migrant women from similar cultural backgrounds. The detailed description of participants, data collection, and analytic processes provided in this study may assist readers to assess the applicability of the findings to their own contexts. Nevertheless, transferability should be considered cautiously given differences in health systems, migrant experiences, and local food environments.

## 5 Conclusion

Building on previous research that identified gaps in existing pregnancy nutrition resources in Australia, this study explored the preferences of women and HCPs for a resource more tailored to the needs of African migrant women when pregnant and their HCP. Both African migrant women and HCPs preferred a combination of electronic and paper‐based resources and discussed the co‐design four potential resources: a mobile application, a website, a pamphlet, and a food guide. Integrating such culturally tailored resources into clinical settings can foster culturally sensitive care, better support women's nutritional needs, and contribute to improved maternal and foetal health outcomes. Action is needed at the national level, with policymakers supporting the development and implementation of culture‐specific nutrition resources that reflect the diversity of Australia's population. Embedding these resources into ANC has the potential to strengthen equity and inclusiveness.

## Author Contributions

**Bolanle R. Olajide:** conceptualisation, investigation, methodology, validation, visualisation, writing – original draft, writing – review and editing, formal analysis. **Paige van der Pligt:** conceptualisation, methodology, validation, formal analysis, supervision, writing – review and editing. **Vidanka Vasilevski:** conceptualisation, methodology, validation, formal analysis, supervision, writing – review and editing. **Fiona H. McKay:** conceptualisation, formal analysis, methodology, validation, supervision, writing – review and editing.

## Ethics Statement

Ethics approval was obtained from the Deakin University Human Research Ethics Committee, with approval number 2023‐331.

## Conflicts of Interest

The authors declare no conflicts of interest.

## Supporting information


**Supporting file 1:** Existing pregnancy nutrition resources.

**Supporting file 2:** Workshop guide.

**Supporting file 3:** Coding tree showing the analytic pathway.

**Supporting file 4:** Culturally tailored features with accompanying participant quotes.

## Data Availability

The authors confirm that the data supporting the findings of this study are available within the article. Further inquiries can be directed at the corresponding author.
