# Supplementary material for: Co‐Designing a Culturally Tailored Nutrition Resource With African Migrant Women and Healthcare Professionals in Australia
Source: Health Expect. 2026 Mar 25;29(2):e70649. doi: 10.1111/hex.70649 (PMC13087440; doi:10.1111/hex.70649)
Supplement: Supplementary file 1 — Supporting file 1: Existing pregnancy nutrition resources. [file HEX-29-e70649-s002.pdf]

HELPFUL HINTS

EATING WELL DURING YOUR PREGNANCY HELPS YOUR BABY DEVELOP AND HAS HEALTH BENEFITS FOR YOU TOO!

Both you and your growing baby need extra nutrients.

Steady weight gain during pregnancy is normal and important for the health of the mother and baby. However, it is also important not to gain too much weight.

So choose foods from the Five Food Groups and limit discretionary foods and drinks high in saturated fat, added sugars and added salt such as cakes, biscuits and potato chips.

You can eat well by:

- Enjoying a variety of fruits and vegetables of different types and colours.
- Increasing your grain consumption to 8–8½ serves a day – mostly wholegrain – in preference to discretionary choices.
- Choosing foods high in iron, such as lean red meat or tofu, which are important for pregnant women.
- Making a habit of drinking milk, eating hard cheese and yoghurt, or calcium enriched alternatives. Reduced fat varieties are best.
- Enjoying a wide variety of vegetables, legumes, fruit and wholegrains and drinking plenty of water every day can assist with constipation – a common occurrence during pregnancy.

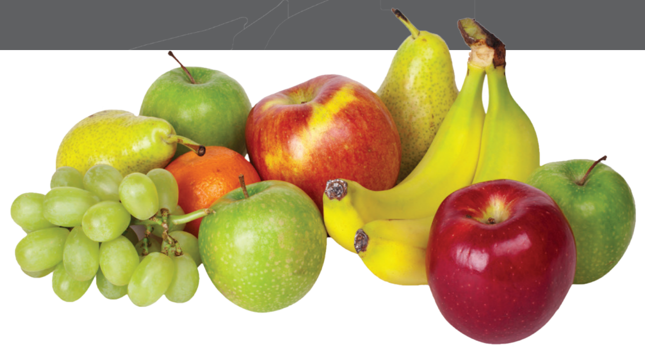

The *Australian Dietary Guidelines* provide up-to-date advice about the amount and kinds of foods that we need to eat for health and wellbeing.

For more information visit:

www.eatforhealth.gov.au

or contact:

National Health and Medical Research Council
GPO Box 1421
Canberra ACT 2601
13 000 NHMRC (13 000 64672)

To order print copies contact:

National Mailing and Marketing
Email: health@nationalmailing.com.au
Phone: 02 6269 1080

Publication Reference: N55h

Australian Government
National Health and Medical Research Council
Department of Health and Ageing

www.eatforhealth.gov.au

Healthy eating during your pregnancy

ADVICE ON EATING FOR YOU AND YOUR BABY

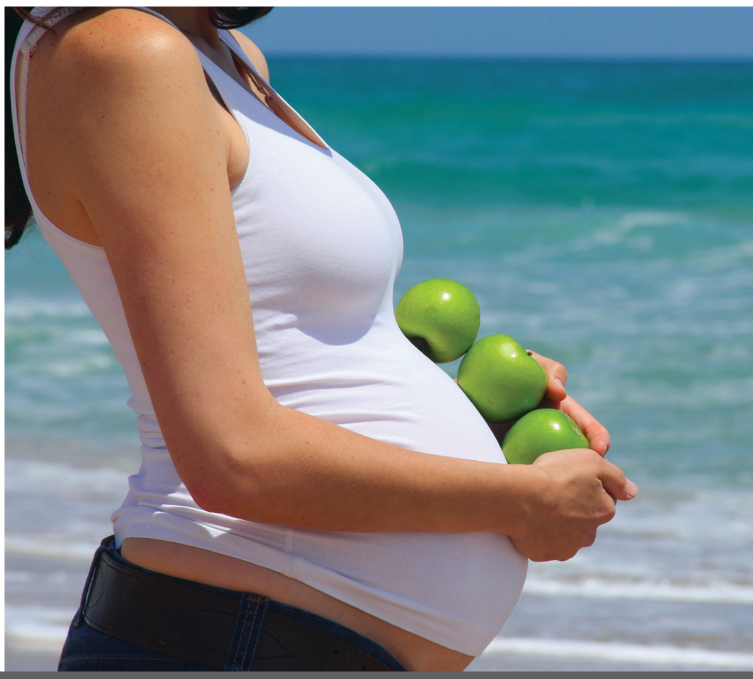

www.eatforhealth.gov.au

WHAT ARE THE DIETARY GUIDELINES?

The *Australian Dietary Guidelines* provide up-to-date advice about the amount and kinds of foods that we need to eat for health and wellbeing. They are based on scientific evidence and research.

The *Australian Dietary Guidelines* of most relevance during pregnancy are included below:

GUIDELINE 1:

To achieve and maintain a healthy weight, be physically active and choose amounts of nutritious food and drinks to meet your energy needs.

GUIDELINE 2:

Enjoy a wide variety of nutritious foods from these five food groups every day:

- Plenty of vegetables of different types and colours, and legumes/beans
- Fruit
- Grain (cereal) foods, mostly wholegrain and/or high cereal fibre varieties, such as breads, cereals, rice, pasta, noodles, polenta, couscous, oats, quinoa and barley
- Lean meats and poultry, fish, eggs, tofu, nuts and seeds, and legumes/beans
- Milk, yoghurt, cheese and/or their alternatives, mostly reduced fat

And drink plenty of water.

Want more information about healthy eating when you are pregnant?

GUIDELINE 3:

Limit intake of foods containing saturated fat, added salt, added sugars and alcohol.

a. Limit intake of foods high in saturated fat such as many biscuits, cakes, pastries, pies, processed meats, commercial burgers, pizza, fried foods, potato chips, crisps and other savoury snacks.
   - Replace high fat foods which contain predominately saturated fats such as butter, cream, cooking margarine, coconut and palm oil with foods which contain predominately polyunsaturated and monounsaturated fats such as oils, spreads, nut butters/pastes and avocado.

b. Limit intake of foods and drinks containing added salt.
   - Read labels to choose lower sodium options among similar foods.
   - Do not add salt to foods in cooking or at the table.

c. Limit intake of foods and drinks containing added sugars such as confectionary, sugar-sweetened soft drinks and cordials, fruit drinks, vitamin waters, energy and sports drinks.

d. For women who are pregnant, planning a pregnancy or breastfeeding, not drinking alcohol is the safest option.

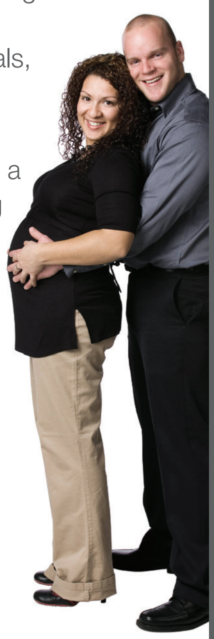

GUIDELINE 4:

Encourage, support and promote breastfeeding.

GUIDELINE 5:

Care for your food; prepare and store it safely.

www.eatforhealth.gov.au

WHICH FOODS SHOULD I AVOID?

PREGNANT WOMEN ARE AT GREATER RISK OF FOOD POISONING AND SHOULD PREPARE AND STORE FOOD CAREFULLY. THEY SHOULD ALSO AVOID ALCOHOL.

Pregnant women should avoid:

- Foods which may contain listeria bacteria like soft cheeses (brie, camembert, ricotta, feta and blue cheese), sandwich meats, bean sprouts, pre-prepared salads and pâté.
- Raw eggs as they may contain salmonella.
- Alcohol – not drinking is the safest option.
- Fish that may contain high levels of mercury – Food Standards Australia New Zealand recommend consuming no more than one serve (100g cooked) per fortnight of shark/flake, marlin or broadbill/ swordfish, and no other fish that fortnight, or one serve (100g cooked) per week of orange roughy (deep sea perch) or catfish and no other fish that week.
- Foods such as nuts during pregnancy only if they are allergic to the foods themselves – avoiding these foods has no impact on the infant's risk of developing allergy symptoms.

Want more information?

www.eatforhealth.gov.au

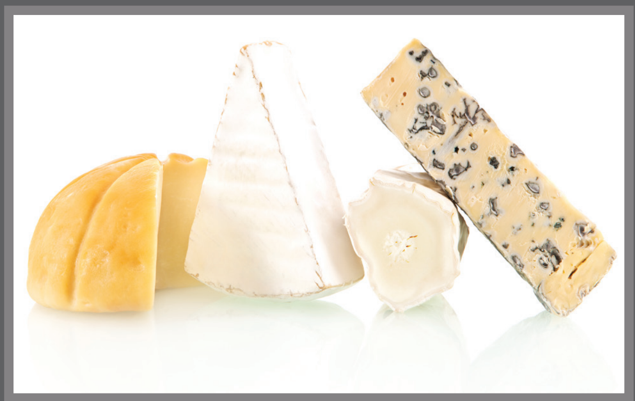

SERVE SIZES

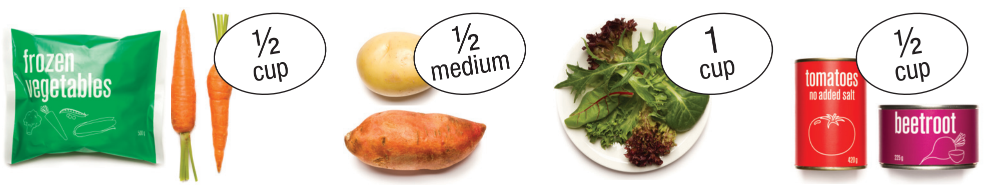

Vegetables and legumes/beans

Serves per day	18 years or under	19–50 years
Women	5	5
Pregnant	5	5
Breastfeeding	5½	7½

A standard serve of vegetables is about 75g (100-350kJ) or:

½ cup	cooked green or orange vegetables (for example, broccoli, spinach, carrots or pumpkin)
½ cup	cooked, dried or canned beans, peas or lentils*
1 cup	green leafy or raw salad vegetables
½ cup	sweet corn
½ medium	potato or other starchy vegetables (sweet potato, taro or cassava)
1 medium	tomato

preferably with no added salt

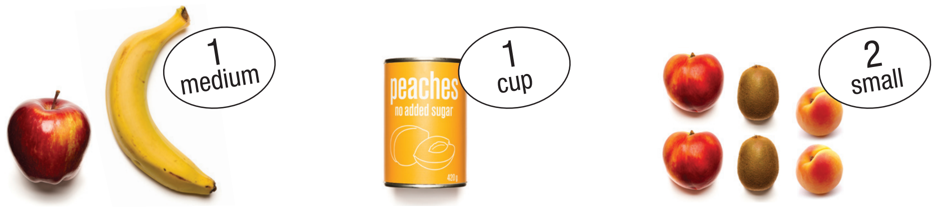

Fruit

Serves per day	18 years or under	19–50 years
Women	2	2
Pregnant	2	2
Breastfeeding	2	2

A standard serve of fruit is about 150g (350kJ) or:

1 medium	apple, banana, orange or pear
2 small	apricots, kiwi fruits or plums
1 cup	diced or canned fruit (with no added sugar)

Or only occasionally:

125ml	(½ cup) fruit juice (with no added sugar)
30g	dried fruit (for example, 4 dried apricot halves, 1½ tablespoons of sultanas)

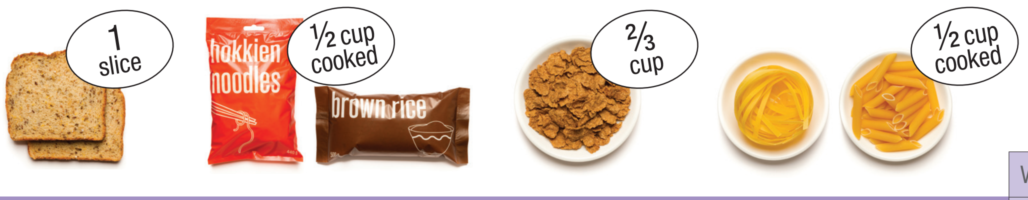

Grain (cereal) foods, mostly wholegrain and/or high cereal fibre varieties

Serves per day	18 years or under	19–50 years
Women	7	6
Pregnant	8	8½
Breastfeeding	9	9

A standard serve (500kJ) is:

1 slice (40g)	bread
½ medium (40g)	roll or flat bread
½ cup (75–120g)	cooked rice, pasta, noodles, barley, buckwheat, semolina, polenta, bulgur or quinoa
½ cup (120g)	cooked porridge
⅔ cup (30g)	wheat cereal flakes
¼ cup (30g)	muesli
3 (35g)	crispbreads
1 (60g)	crumpet
1 small (35g)	English muffin or scone

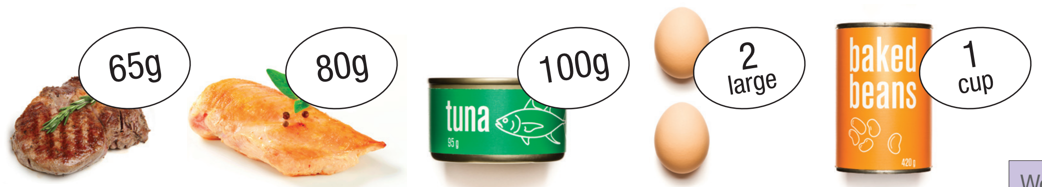

Lean meats and poultry, fish, eggs, tofu, nuts and seeds, and legumes/beans

Serves per day	18 years or under	19–50 years
Women	2½	2½
Pregnant	3½	3½
Breastfeeding	2½	2½

A standard serve (500-600kJ) is:

65g	cooked lean meats such as beef, lamb, veal, pork, goat or kangaroo (about 90–100g raw)*
80g	cooked lean poultry such as chicken or turkey (100g raw)
100g	cooked fish fillet (about 115g raw weight) or one small can of fish
2 large (120g)	eggs
1 cup (150g)	cooked or canned legumes/beans such as lentils, chick peas or split peas (preferably with no added salt)
170g	tofu
30g	nuts, seeds, peanut or almond butter or tahini or other nut or seed paste (no added salt)

weekly limit of 455g

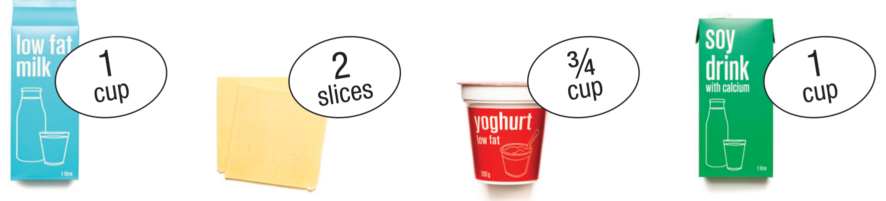

Milk, yoghurt, cheese and/or alternatives, mostly reduced fat

Serves per day	18 years or under	19–50 years
Women	3½	2½
Pregnant	3½	2½
Breastfeeding	4	2½

A standard serve (500-600kJ) is:

1 cup (250ml)	fresh, UHT long life, reconstituted powdered milk or buttermilk
½ cup (120ml)	evaporated milk
2 slices (40g)	or 4 x 3 x 2cm cube (40g) of hard cheese, such as cheddar
¾ cup (200g)	yoghurt
1 cup (250ml)	soy, rice or other cereal drink with at least 100mg of added calcium per 100ml

- **To meet additional energy needs, extra serves from the Five Food Groups or unsaturated spreads and oils, or discretionary choices may be needed only by those women who are taller or more active, but not overweight.**
- **An allowance for unsaturated spreads and oils for cooking, or nuts and seeds can be included in the following quantities: 14–20g per day for pregnant and breastfeeding women.**
- **For meal ideas and advice on how to apply the serve sizes go to:**

www.eatforhealth.gov.au

FOR FURTHER INFORMATION GO TO www.eatforhealth.gov.au

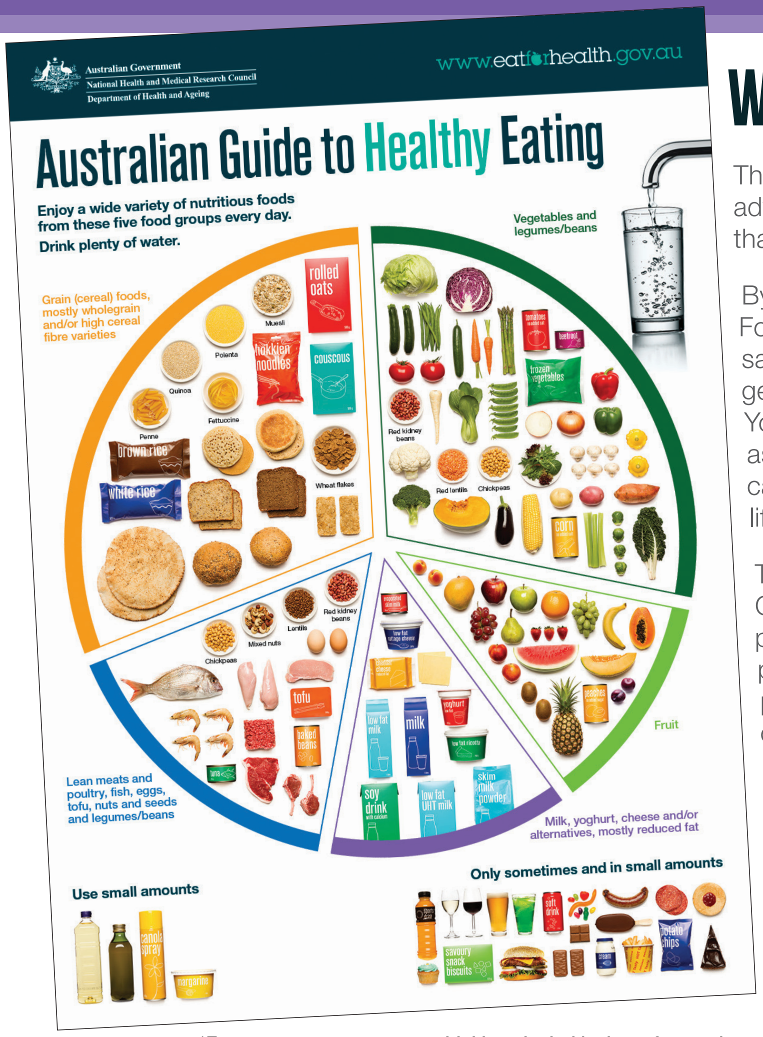

*For pregnant women – not drinking alcohol is the safest option.

WHICH FOODS SHOULD I EAT AND HOW MUCH?

The *Australian Dietary Guidelines* provide up-to-date advice about the amount and kinds of foods and drinks that we need regularly, for health and well-being.

By eating the recommended amounts from the Five Food Groups and limiting the foods that are high in saturated fat, added sugars and added salt, you will get enough of the nutrients essential for good health. You may reduce your risk of chronic diseases such as heart disease, type 2 diabetes, obesity and some cancers. You may also feel better, look better, enjoy life more and live longer!

The amount of food you will need from the Five Food Groups depends on your age, height, weight and physical activity levels, and also whether you are pregnant or breastfeeding. For example, if you're pregnant you should aim to eat at least 8½ serves of grain (cereal) foods a day. You might notice that the number of serves you need from the Five Food Groups changes when you are pregnant or breastfeeding – this is due to changes in your nutrient requirements for your growing baby's needs and to support breastfeeding.

For further information go to www.eatforhealth.gov.au.

HOW MUCH IS A SERVE?

It's helpful to get to know the recommended serving sizes and serves per day so that you eat and drink the right amount of the nutritious foods you and your baby need for health – as shown in the tables above. We've given you the serve size in grams too, so you can weigh foods to get an idea of what a serve looks like.

The 'serve size' is a set amount that doesn't change. It is used along with the 'serves per day', to work out the total amount of food required from each of the Five Food Groups. 'Portion size' is the amount you actually eat and this will depend on what your energy needs are. Some people's portion sizes are smaller than the 'serve size' and some are larger. This means some people may need to eat from the Five Food Groups more often than others.

HOW MANY SERVES A DAY?

Few people eat exactly the same way each day and it is common to have a little more on some days than others. However, on average, the total of your portion sizes should end up being similar to the number of serves you need each day.

If you eat portions that are smaller than the 'serve size' you will need to eat from the Food Groups more often. If your portion size is larger than the 'serve size', then you will need to eat from the Food Groups less often.

Nutrition + Pregnancy

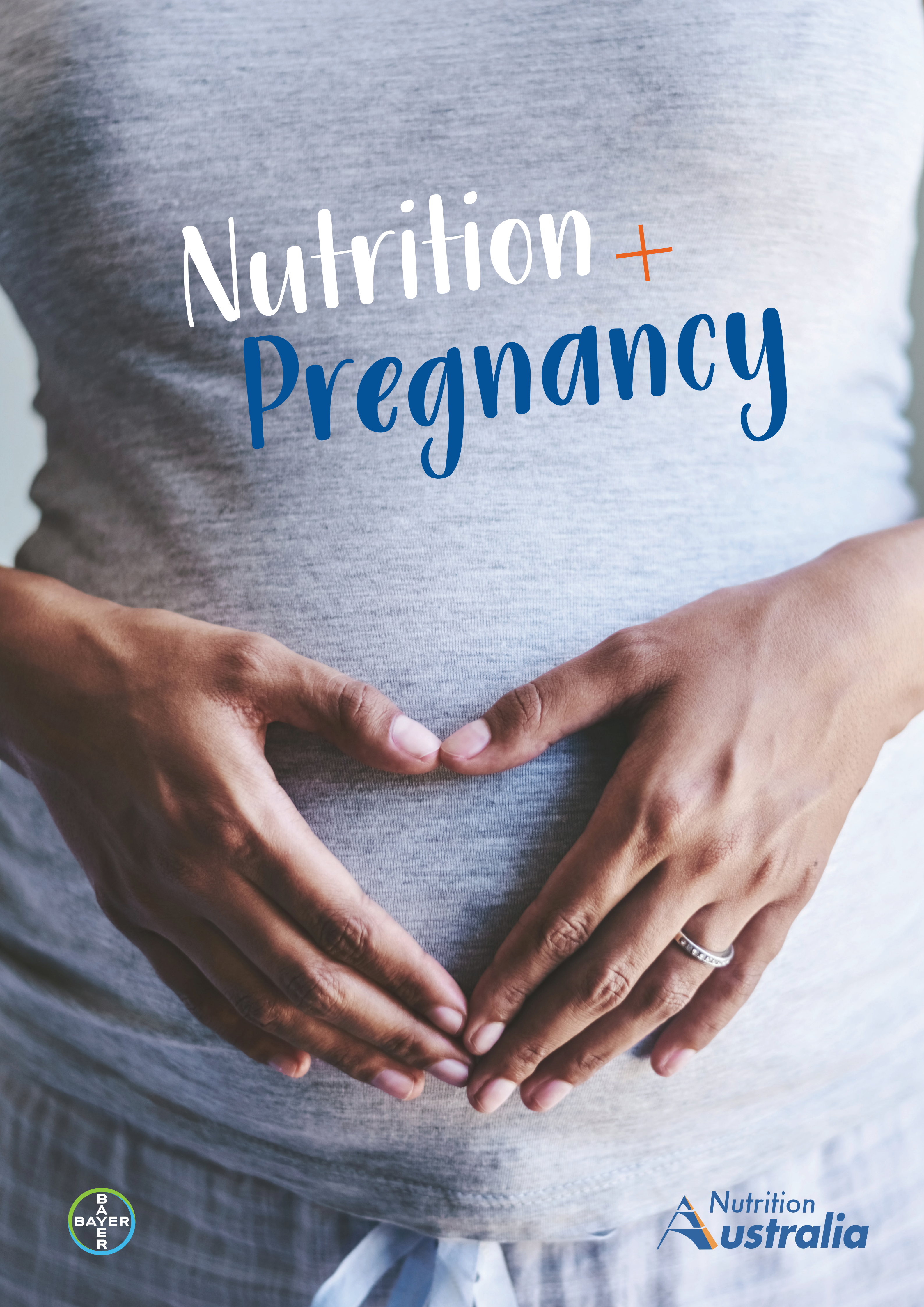

Nutrition Australia, in collaboration with Bayer Australia, has developed the following guide for women during pregnancy, outlining dietary requirements to ensure they receive adequate nutrition for themselves and their developing baby.

This guide will help pregnant women understand the recommended Australian dietary requirements, encouraging healthy choices. Within, you will find current information to support good nutrition during pregnancy, providing easy to use advice including suggested meal and snack ideas.

Evidence shows that establishing good nutrition during the first 1000 days of life[1] (starting from conception) can positively influence life-long health. However, pregnancy is a nutritionally demanding time, and many Australian women may not be getting all the nutrients they need during pregnancy.

For instance, recent research[2] showed that while 61% of women surveyed online believed their diet was healthy during pregnancy, not one woman reported meeting the recommendations for all five food groups. This is why we have developed this guide as an easy reference source for pregnant women and health professionals alike.

Nutrition Australia

BAYER

References

1 Moore, Tim & Arefadib, Noushin & Deery, Alana & West, Sue, MA & Royal Children's Hospital (Melbourne, Vic.). Centre for Community Child Health (2017). The first thousand days : an evidence paper. Centre for Community Child Health, Royal Children's Hospital Melbourne, Parkville, Vic

2 Malek L, Umberger W, Makrides M, Zhou SJ. Adherence to the Australian dietary guidelines during pregnancy: evidence from a national study. Public Health Nutr 2015; 19(7):1155-6

Contents

Energy needs during pregnancy 3
Healthy snack examples 4
Quality vs quantity of food 5
What to eat and how much each day 6
Maintaining a healthy weight during pregnancy 7
Vitamin and mineral supplementation for pregnancy 8
Food safety 10
The foods to avoid... 11
and what you can replace them with 12
Mercury 13
FAQ 14

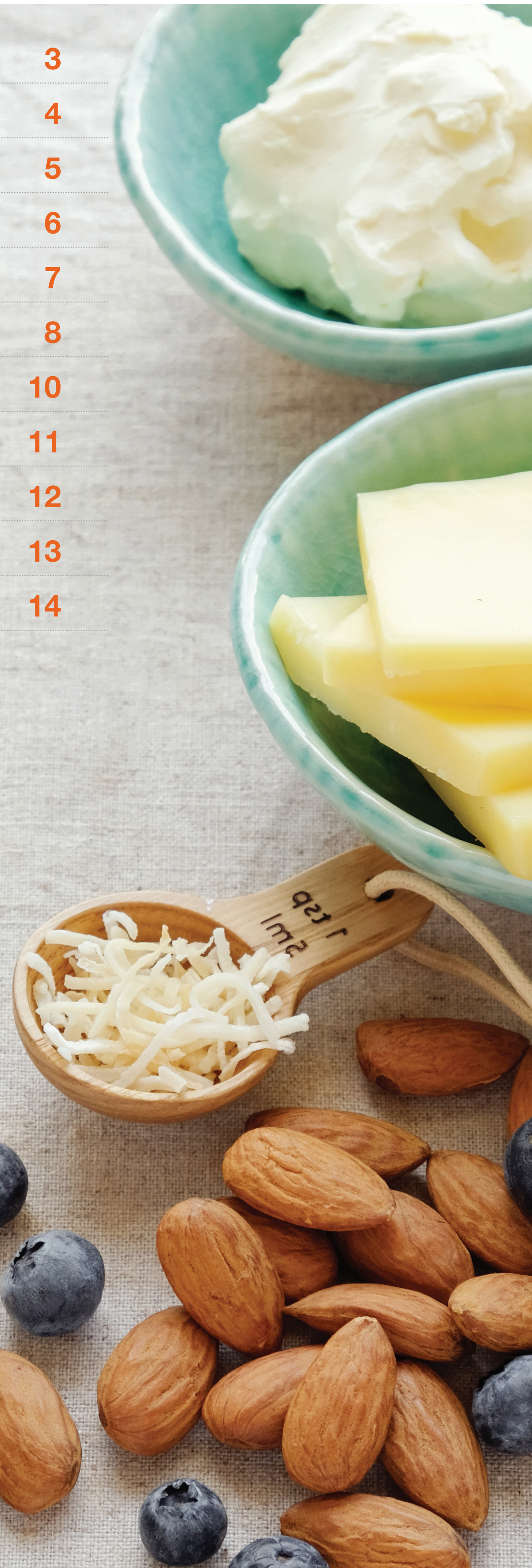

Energy needs during pregnancy

Pregnancy is such a special time in a woman's life. We know that the food you eat while pregnant affects your own health and wellbeing and the health of your developing baby. There is a lot of conflicting advice around what you should and shouldn't eat during pregnancy, and it can feel overwhelming. We hope to clear up any confusion for you, so you can enjoy a happy, healthy and stress-free pregnancy!

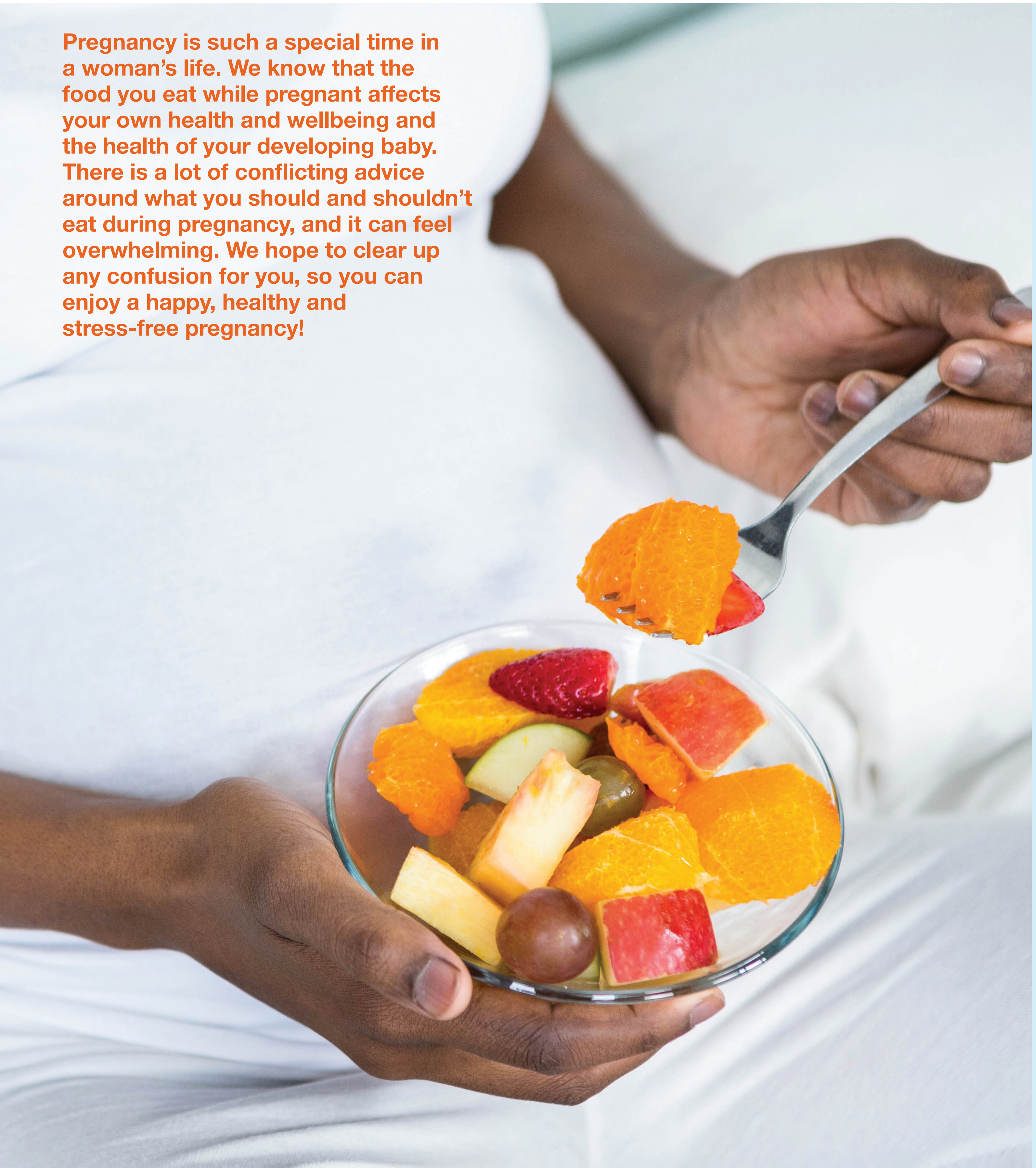

Healthy snack examples include:

How often have you been told that you are eating for two while pregnant? While you hear this a lot, you actually do not need to eat twice the amount of food during pregnancy.

In fact, during the first 3 months (first trimester) of pregnancy you do not need any extra kilojoules on top of your normal diet.

During the second and third trimester of your pregnancy you do need to increase your daily kilojoule intake, but probably by less than you imagine.

A couple of extra snacks across the day can be an easy way to get these additional kilojoules, especially towards the end of pregnancy when many women find it difficult to eat large meals, due to reflux or feeling full very quickly.

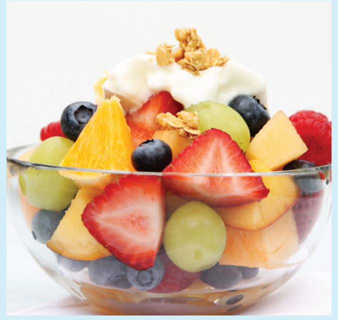

Sliced fresh fruit with a few spoons of yoghurt on top

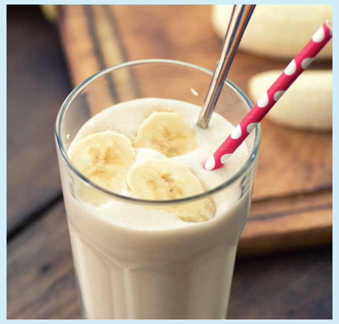

A fruit smoothie made with fresh fruit, milk, yoghurt and rolled oats

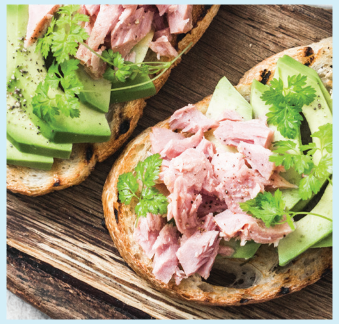

A small tin of tuna with wholegrain crackers and avocado

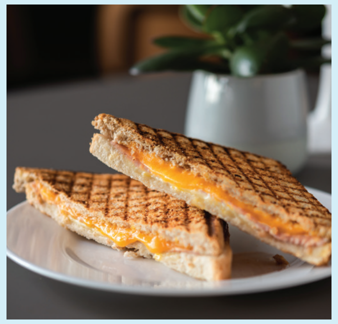

A toasted wholegrain sandwich, filled with a small tin of baked beans and cheddar cheese

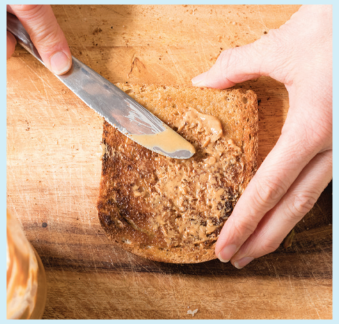

Wholegrain toast topped with peanut butter

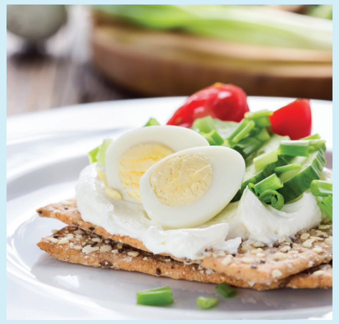

Sliced hard-boiled eggs on wholegrain crispbreads

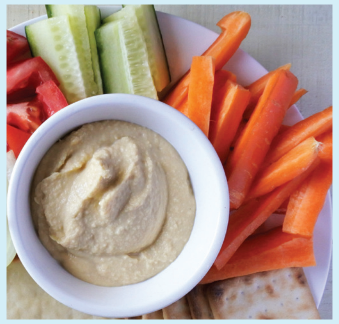

Hummus dip and vegetable sticks

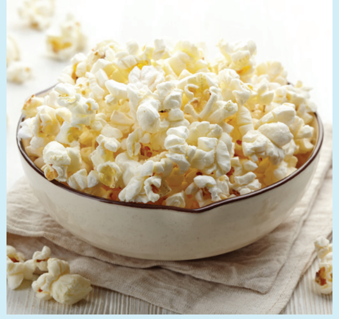

Air popped popcorn

Quality vs quantity of food

There is a lot of focus on the quantity of food during pregnancy, however you should be paying most attention to the nutritional quality of the foods you are eating.

During pregnancy you require more of certain vitamins and minerals – up to 50% for some nutrients, so choosing nutrient-rich foods that are high in vitamins and minerals is essential.

Let's use a morning tea snack as an example of choosing nutrient-rich foods. For morning tea you could either have a piece of fresh fruit, or 2 plain milk arrowroot biscuits. These two snack options contain roughly the same number of kilojoules, so in terms of quantity they are pretty much equal.

When we consider quality however these two options are very different. By choosing a piece of fruit to snack on, you will also be getting a good dose of dietary fibre and vitamins such as vitamin C and folate. In contrast, sweet biscuits are not a good source of key nutrients, as they contain very little fibre and vitamins.

We recommend basing your meals and snacks on the core food groups each day. Occasional treats are fine, but eating a variety of foods from the core food groups will help to ensure you get all the nutrients you and your developing baby need.

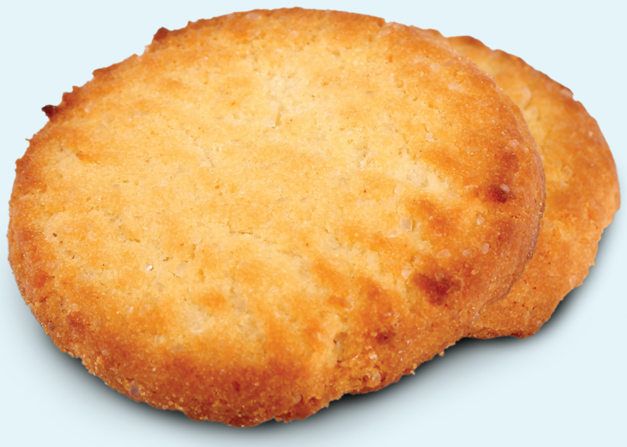

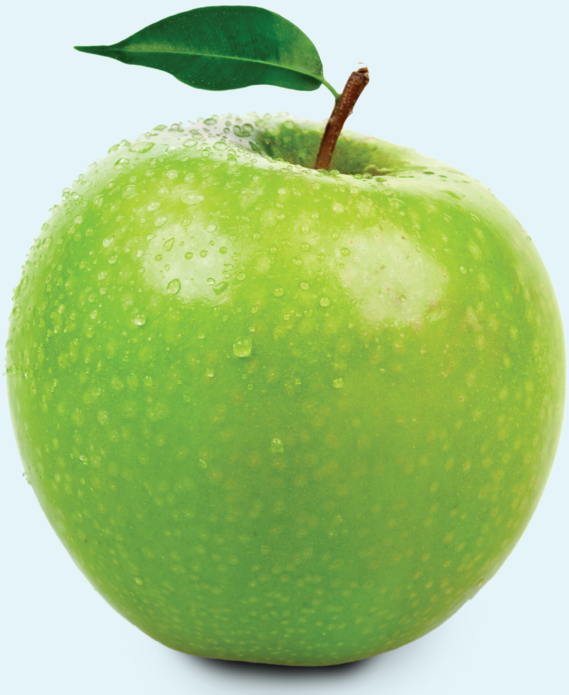

What to eat and how much each day

The following table shows the number of serves each day from each food group for pregnant women aged 19-50 years*.

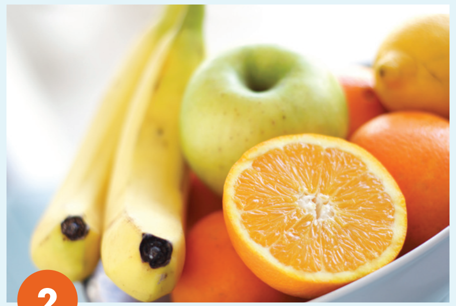

2 serves of fruit

- 1 medium apple, banana, orange or pear
- 2 small apricots, kiwi fruits or plums
- 1 cup diced or canned fruit (with no added sugar)

Occasionally

- 125ml or ½ cup fruit juice with no added sugar
- 30g dried fruit (i.e. 4 dried apricots, or 1 ½ tablespoons of sultanas)

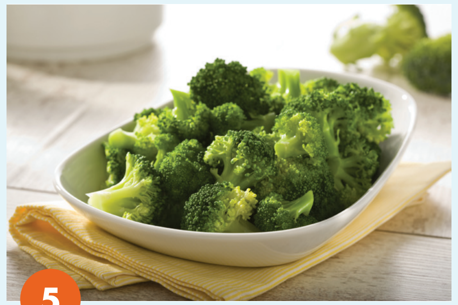

5 serves of vegetables

- ½ cup cooked vegetables i.e. broccoli, spinach, carrots, pumpkin
- ½ cup cooked, dried or canned beans, peas, lentils
- 1 cup green leafy or raw salad vegetables
- ½ cup sweet corn
- ½ medium potato
- 1 tomato

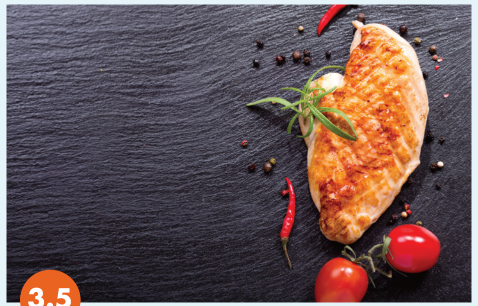

3.5 serves of meats and alternatives

- 65g cooked lean meats such as beef, lamb, veal, pork (90-100g raw)
- 80g cooked lean poultry such as chicken or turkey (100g raw)
- 100g cooked fish fillet or one small can of fish (refer to guidelines in page 13 regarding mercury)
- 2 eggs
- 1 cup cooked or canned legumes/beans such as lentils, chickpeas or split peas
- 30g nuts, seeds, peanut or almond butter, tahini
- 170g tofu

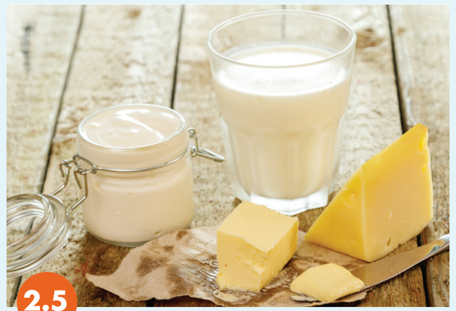

2.5 serves of dairy

- 1 cup (250ml) milk
- 1 cup (250ml) soy, rice almond or other cereal-based milk,with at least 100mg of added calcium per 100ml
- ¾ cup (200g) yoghurt
- 2 slices or 40g hard cheese i.e. cheddar

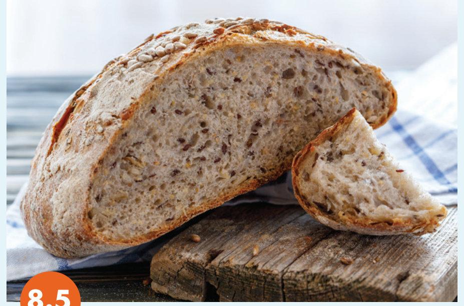

8.5 serves of grains

- 1 slice bread
- ½ medium bread roll or flat bread/wrap
- ½ cup cooked rice, pasta, noodles, buckwheat, polenta, semolina, quinoa
- ½ cup cooked porridge
- 2/3 cup wheat cereal flakes
- ¼ cup muesli
- 1 small English muffin or scone.
- 3 crispbreads
- 1 crumpet

*Pregnant women aged 18 years and under require 3.5 serves of dairy daily and 8 serves of grains daily.

Maintaining a healthy weight during pregnancy

It is difficult to know how much weight to expect to gain during pregnancy. However, maintaining a healthy weight is very important for you and your baby both in the short and long term. It is best to talk to your GP, obstetrician or healthcare professional, as every woman is different and they will be able to give you individual advice. Generally speaking, the ideal weight gain during pregnancy depends on your pre-pregnancy body mass index (BMI), as shown below. If you are unsure what your pre-pregnancy BMI is, your doctor can calculate this for you.

Try to remember pregnancy is not a time for strict 'dieting'. If you or your health professional believe you are gaining too much weight there are ways you can manage this without depriving yourself of food. For example, you could introduce some moderate exercise (with advice from your doctor or other health professional).

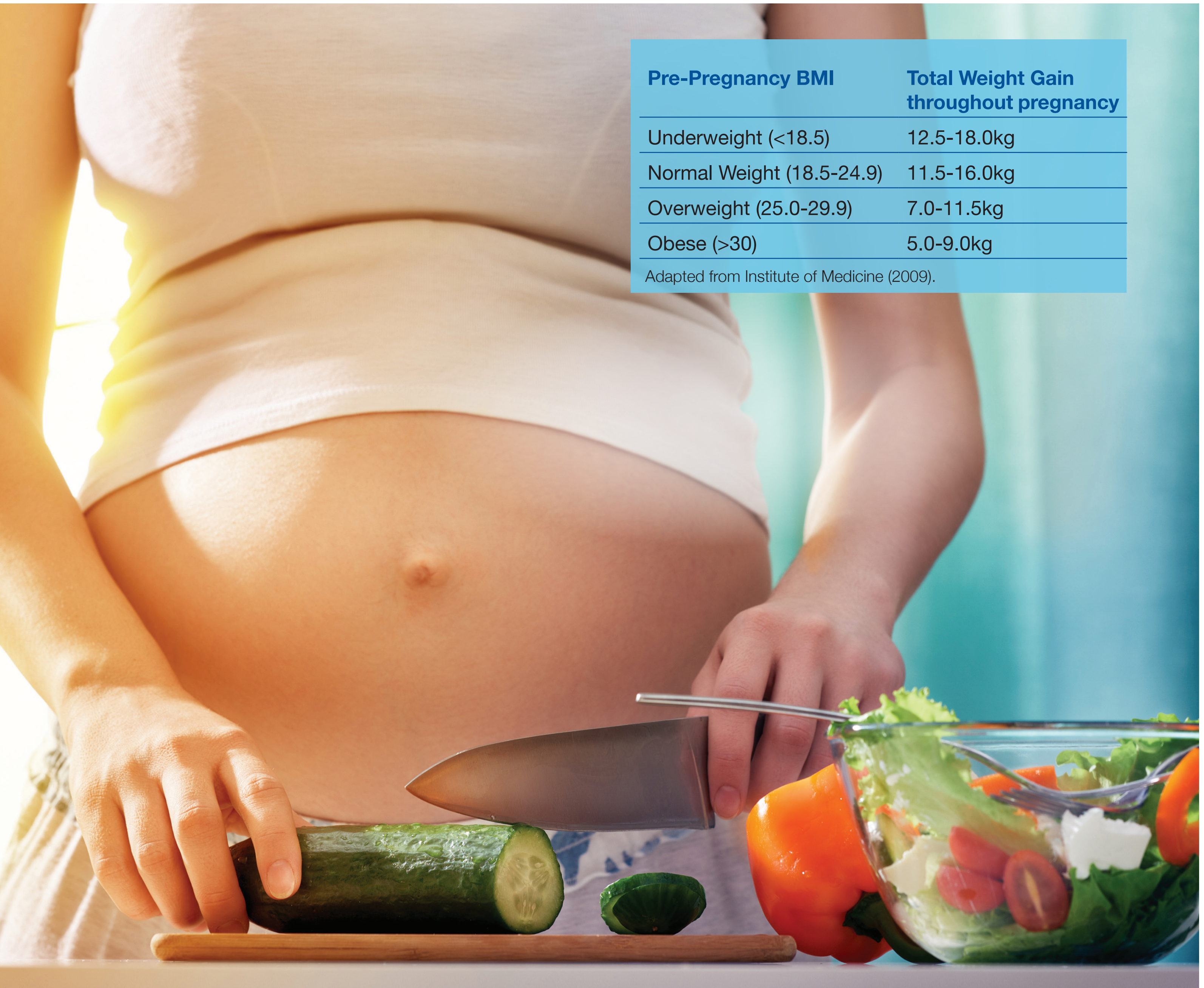

Pre-Pregnancy BMI	Total Weight Gain throughout pregnancy
Underweight (<18.5)	12.5-18.0kg
Normal Weight (18.5-24.9)	11.5-16.0kg
Overweight (25.0-29.9)	7.0-11.5kg
Obese (>30)	5.0-9.0kg

Adapted from Institute of Medicine (2009).

Vitamin and mineral supplementation for pregnancy

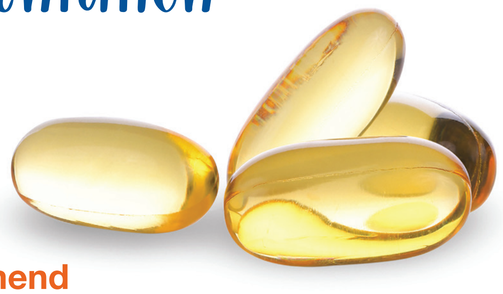

Even when choosing nutritious foods, it can be difficult to get enough of all the essential nutrients you need from your diet alone. Therefore we recommend taking a pregnancy multivitamin supplement each day in addition to a healthy diet.

Why is it important?

A daily pregnancy multivitamin is not a replacement for a healthy diet; rather it supports a healthy diet to help you meet your increased nutrition needs during pregnancy. There are many key nutrients that have an important role during pregnancy, and many that you require more of during this time.

Folate:

Folate is a B-vitamin which is particularly important prior to conception and during the first trimester of pregnancy. Folate has an essential role in the formulation of your baby's neural tube in the very early weeks of pregnancy. The neural tube later develops into the brain and spine. Foods that are high in folate include green leafy vegetables, legumes, fruit, and bread/cereals, so try to include these foods in your diet. Australian Guidelines recommend taking a folic acid-containing supplement (or a pregnancy multivitamin containing folate) at least four weeks before you start trying for a baby. This will ensure your folate levels are high enough to help protect against neural tube defects.

Reference: The Royal Australian and New Zealand College of Obstetricians and Gynaecologists.

Iodine:

Iodine is another important nutrient, as mild to moderate deficiency during pregnancy can impact your baby's hearing and physical development, and has been linked to learning difficulties. Foods high in iodine include seafood (but be mindful of the seafood to avoid and those to have in moderation during pregnancy as discussed on page 13), and bread. In addition to eating foods high in iodine, it is recommended that women who are planning a pregnancy, pregnant, or breastfeeding take an iodine supplement daily (or a pregnancy multivitamin containing iodine).

Reference: The Royal Australian and New Zealand College of Obstetricians and Gynaecologists.

Iron:

You require 50 percent more iron during pregnancy, which can be hard to obtain from your diet alone. Your doctor will monitor your iron levels closely, as iron deficiency during pregnancy is actually quite common. Include plenty of iron-rich foods such as red meats, green leafy vegetables and grains in your diet daily. Your pregnancy multivitamin will also help you to meet your iron requirement.

Vitamin D:

Vitamin D is another important nutrient during pregnancy, and many women may actually have low levels prior to becoming pregnant. Some groups of women may require additional supplementation, for instance women with dark or veiled skin or sun-avoidant office workers. Discuss this with your doctor, as they will be able to monitor your levels and determine whether you need supplementation.

When to start taking the supplement

We recommend you start a pregnancy multivitamin daily 1 month prior to conception, or as soon as you find out you are pregnant, and continue this for your entire pregnancy. If you experience nausea and/or vomiting, take your supplement at the time of day you feel the best.

Breastfeeding is also a demanding time, during which women require additional nutrition. We recommend you speak to your doctor or healthcare professional about your new needs once your baby is born. This may include continuing to take a supplement to help ensure you are getting enough nutrients.

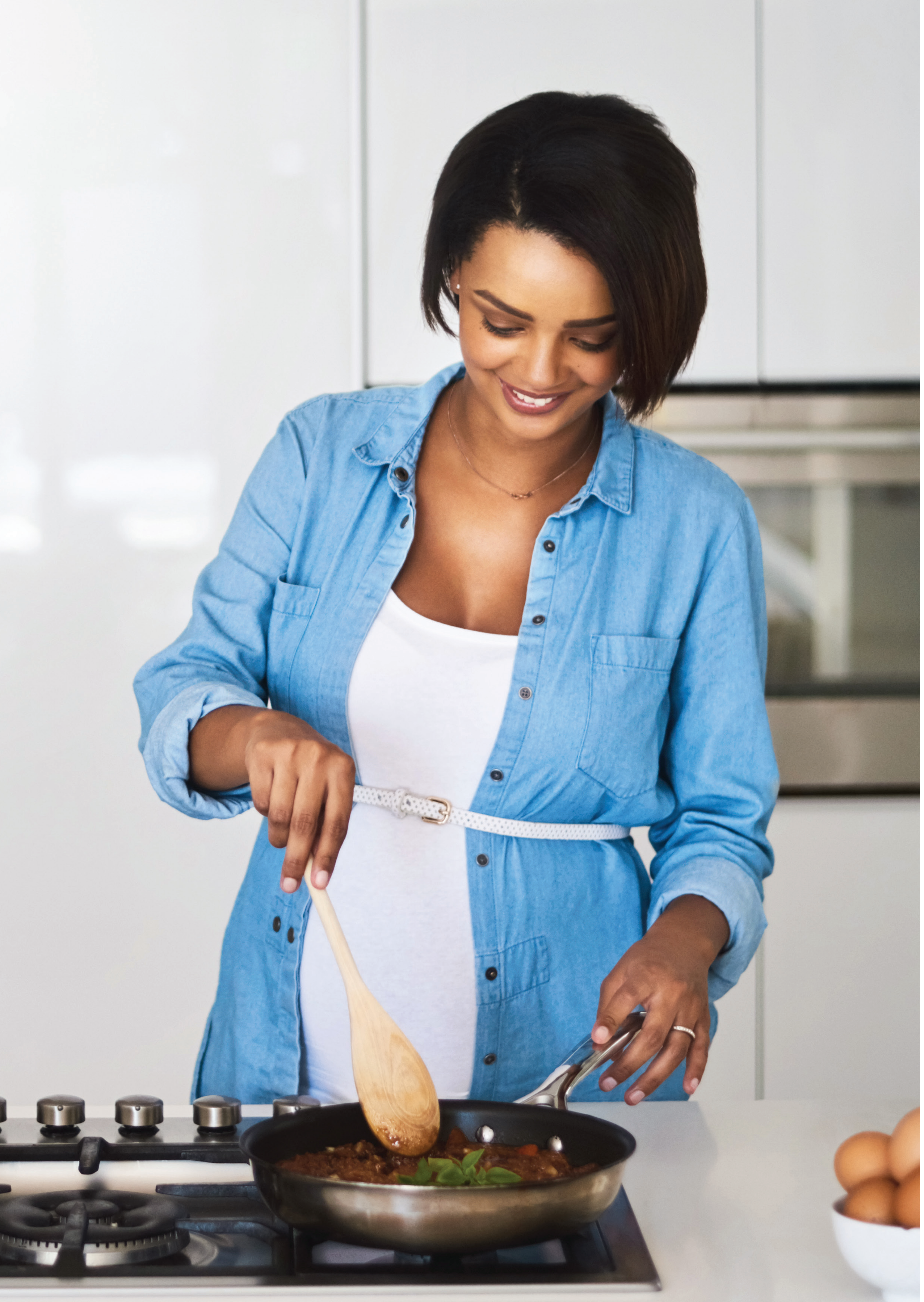

Food safety

It is particularly important to take special care when preparing and storing your food during pregnancy. There are also certain foods which must be avoided.

There are hormonal changes that occur during pregnancy that lower your immune system. This makes it more difficult for your body to fight off illness and infection, placing you at greater risk of food poisoning. Many women understandably feel quite stressed about this and there is a lot of conflicting information available. You can lower your risk by following good food safety practices and avoiding certain high-risk foods.

To help you ensure your food is safe, we've outlined the risks to be aware of, foods to avoid and suitable replacement options (see following page).

Listeria:

Listeria is a type of bacteria found in certain foods. It can cause a serious infection called Listeriosis, which may only cause mild symptoms in the mother but can have devastating effects on an unborn child, with some cases resulting in miscarriage, still birth and premature birth.

Salmonella:

Salmonella food poisoning can cause serious illness, with severe nausea and vomiting sometimes resulting in miscarriage. Raw or undercooked, runny eggs and undercooked meats can contain salmonella, so it is important to avoid these.

Toxoplasmosis:

Toxoplasmosis is an infection caused most commonly by touching cat faeces (i.e. when cleaning the cat litter), or from contaminated soil in the garden. Often the infection does not cause any symptoms at all, but exposure in pregnancy can be serious. You can also be at risk from consuming undercooked or raw meat. Wear gloves when gardening and minimise contact with cats and cat litter.

General good food safety practices to follow:

- Wash fruits and vegetables well.
- Wash hands before preparing food, and ensure all preparation surfaces and utensils are cleaned thoroughly.
- Keep leftovers in the refrigerator only until the next day, and reheat until food is steaming hot.
- Do not eat food that is past its 'use by' or 'best before' dates.

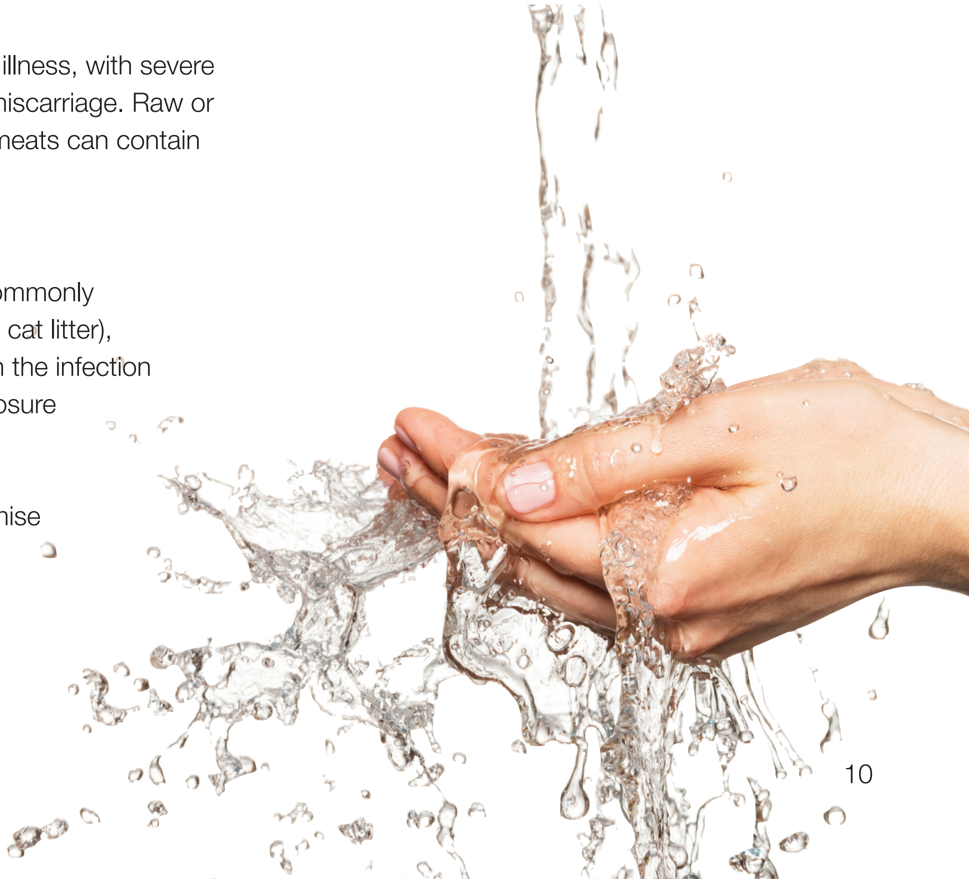

The foods to avoid... and what you can replace them with:

If you have any doubts about a food, or are concerned whether it has been stored and prepared safely or not – then best to avoid it and not take the risk!

	FOOD TO AVOID		SAFER ALTERNATIVES
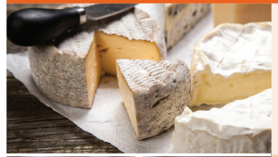	Soft cheeses, i.e. brie, camembert, feta, ricotta, blue cheese, dips that include these cheeses.	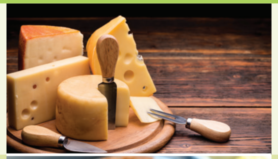	Hard cheeses i.e. cheddar. Soft cheeses are safe in dishes that are cooked to steaming hot and eaten straight away i.e. spinach and ricotta cannelloni.
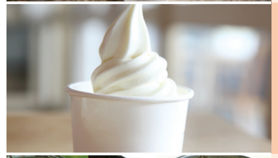	Soft serve ice cream.	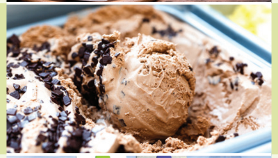	Store-bought ice cream that is stored correctly in the freezer.
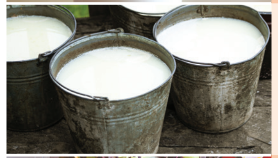	Unpasteurised dairy products.	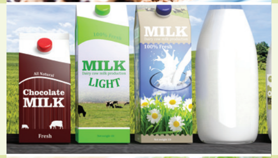	Pasteurised dairy products.
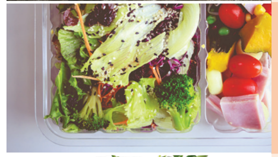	Pre-packaged salads and fruit, including from sandwich/salad bars and buffets.	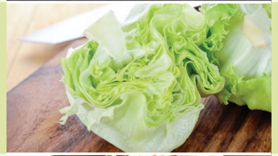	Buy whole lettuces and wash and prepare yourself at home. Buy fresh fruit and make your own fruit salad at home.
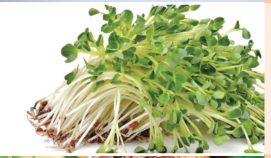	Sprouted seeds such as alfalfa, bean and snow pea sprouts.	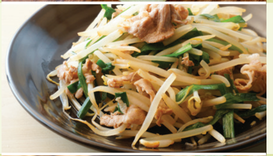	Sprouts that have been thoroughly cooked through in dishes.
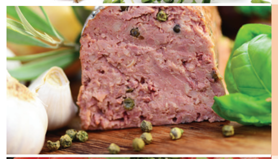	Paté, meat or fish pastes.	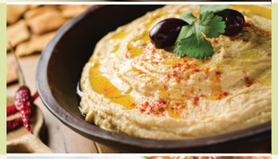	Hummus dip, tinned tuna or salmon.
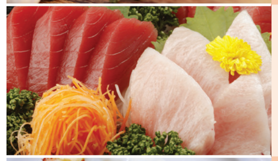	Chilled seafood i.e. oysters, sashimi and sushi, smoked ready-to-eat seafood, and cooked ready-to-eat prawns.	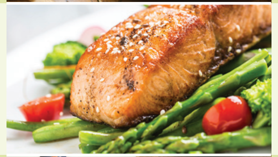	Freshly cooked seafood and fish in keeping with the recommendations on mercury on page 13. Canned seafood (including tuna, salmon and sardines).
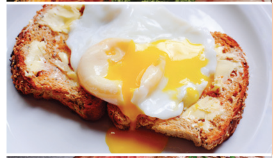	Raw or undercooked eggs and items containing these i.e. a runny poached egg, homemade or café mayonnaise/aioli, desserts containing raw eggs.	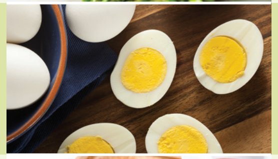	Hard boiled eggs, scrambled eggs. Store bought mayonnaise/aioli and follow storage instructions Baked cakes, muffins and desserts etc.
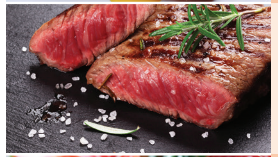	Rare or medium-cooked meats.	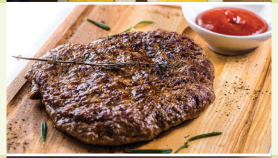	Meat cooked all the way through.
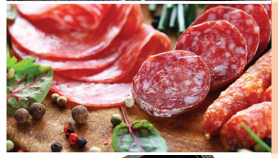	Cold deli meats from delicatessens, sandwich bar or buffets and sliced ready-to-eat packaged deli meats i.e. salami, ham.	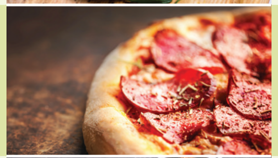	These meats are safe if cooked thoroughly until steaming hot, and eaten straight away i.e. on a pizza. Do not eat cold.
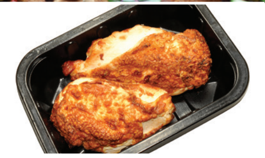	Cold cooked ready-to-eat chicken.	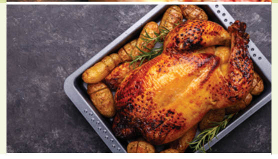	Whole roast chicken, freshly cooked and eaten straight away while hot. Do not eat the stuffing.

Mercury

Fish contains many nutrients including protein, healthy fats and iodine, making it a nutrient-rich food. During pregnancy however, it is important to be mindful of eating too much of certain types of fish that are high in mercury. Mercury is a naturally-occurring element that is present in seafood. If you consume too much, it can build up in your system and affect your baby's developing nervous system.

Certain fish have higher levels of mercury than others. We recommend that you enjoy a serve of freshly cooked fish **2-3 times a week**, while sticking to the below recommendations.

Pregnant Women 1 serve = 150g				
2-3 serves per week of any fish and seafood not listed at right	OR	1 serve per week of orange roughy (deep sea perch) or catfish, and no other fish that week	OR	1 serve per fortnight of flake (shark) or billfish (swordfish/ broadbill and marlin) and no other fish that fortnight

Food Standards Australia and New Zealand.

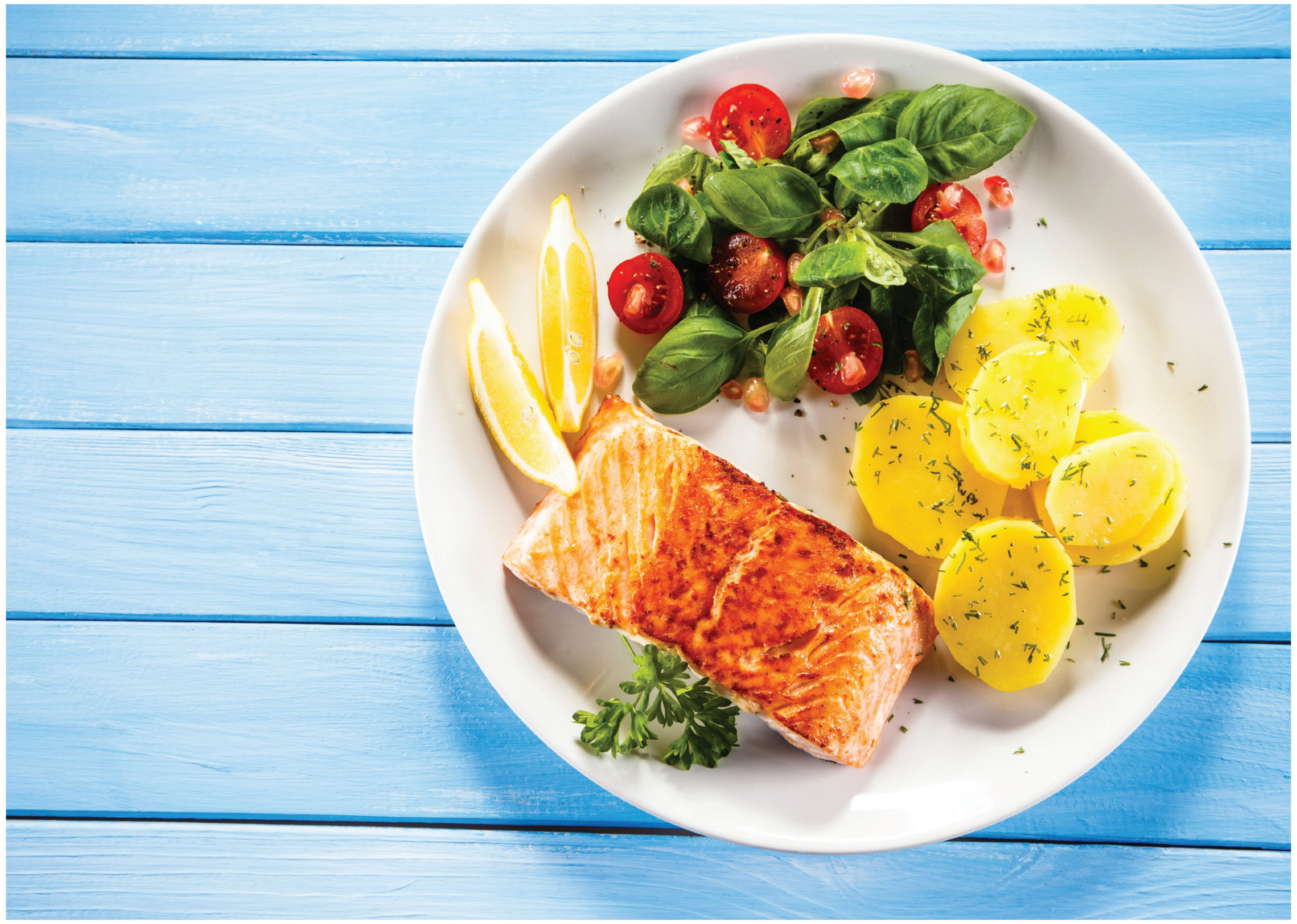

FAQ

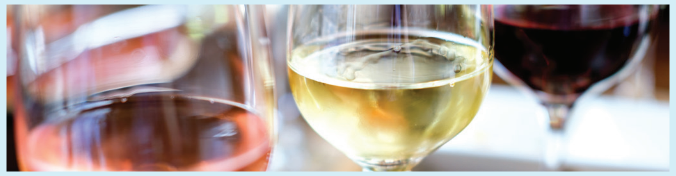

1. Can I drink any alcohol during pregnancy?

We recommend not consuming any alcohol while pregnant, as this is the safest option for you and your baby.

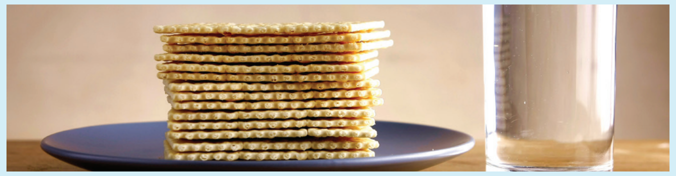

2. Are there any foods that might help manage my morning sickness?

The following strategies might help ease your morning sickness symptoms:

- Try having smaller, more frequent meals throughout the day. Avoid large meals and very spicy or greasy foods.
- Keep some dry crackers and a glass of water next to your bed. Eat those and sip water before getting out of bed.
- Some women find drinking fluids easier to keep down than a meal. Try making smoothies and sip slowly.
- Remember to drink plenty of water. Ginger tea may also be helpful.
- Stay away from strong food smells and use the exhaust fan when cooking to minimise these.
- If you are finding your symptoms difficult to manage, speak with your pharmacist or doctor. There are medicines available (including without a prescription) that can help ease your symptoms.

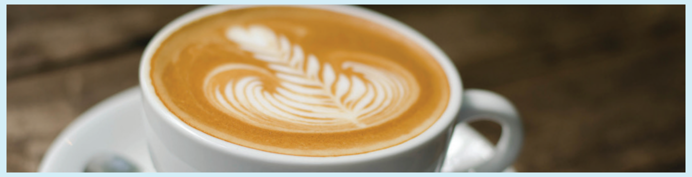

3. How much caffeine is safe to have per day?

Caffeine is safe to drink in moderate amounts. Discuss with your healthcare professional for individual advice regarding an appropriate caffeine intake for you as this can vary. Generally depending on the strength of the drink, 1-2 espresso style coffees, or 2-3 cups of instant coffee or 3-5 cups of tea is considered safe. Choose decaffeinated options when possible and be mindful of the caffeine content of certain soft drinks. We recommend avoiding all energy drinks.

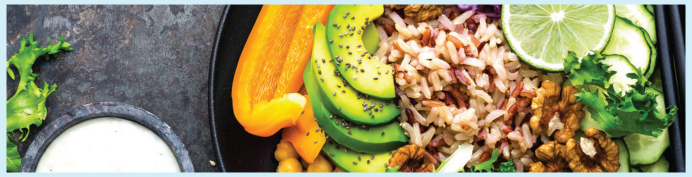

4. What if I follow a vegetarian or vegan diet? How do I make sure I get all the nutrients I need?

If you follow a vegetarian or vegan diet you may require additional supplements along with your daily pregnancy multivitamin. It is best to discuss this with your doctor or dietitian as they will determine what supplementation is appropriate for your individual needs.

References

National Health and Medical Research Council, Australian Government Department of Health and Ageing, New Zealand Ministry of Health. Nutrient Reference Values for Australia and New Zealand. Canberra: National Health and Medical Research Council; 2006. Available at: https://www.nrv.gov.au/

National Health and Medical Research Council (2013) Australian Dietary Guidelines. Canberra: National Health and Medical Research Council. Available at: https://www.eatforhealth.gov.au/

The Royal Australian and New Zealand College of Obstetricians and Gynaecologists. Vitamin and mineral supplementation and pregnancy. East Melbourne, Vic: RANZCOG, 2015 [Accessed Dec 2018]

Food Standards Australia and New Zealand (FSANZ). Available at: http://www.foodstandards.gov.au

IOM (Institute of Medicine) and NRC (National Research Council). 2009. Weight Gain During Pregnancy: Re-examining the Guidelines. Washington, DC: The National Academies Press. Available at: https://www.ncbi.nlm.nih.gov/books/NBK32813/pdf/Bookshelf_NBK32813.pdf

NSW Government Food Authority. Available at: http://www.foodauthority.nsw.gov.au/foodsafetyandyou/life-events-and-food/pregnancy/foods-to-eat-or-avoid-when-pregnant

Victoria State Government. Available at: https://www2.health.vic.gov.au/about/publications/factsheets/Listeria---the-facts

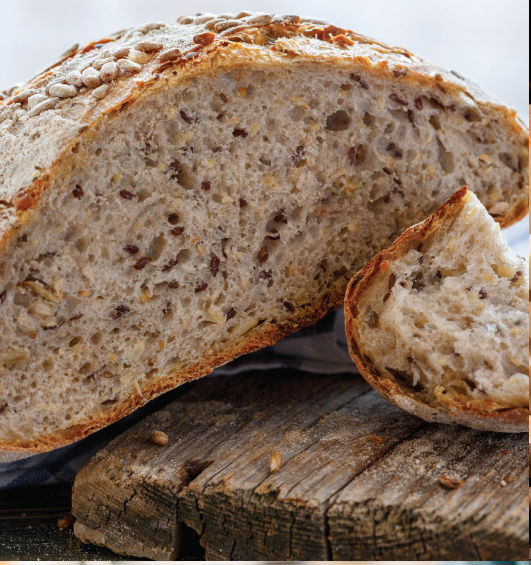
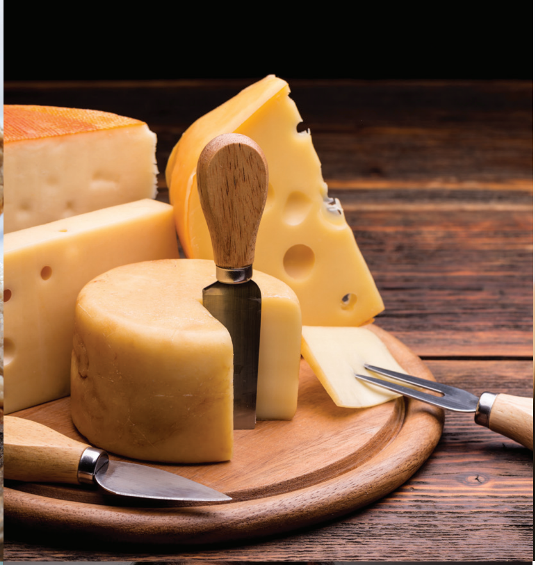
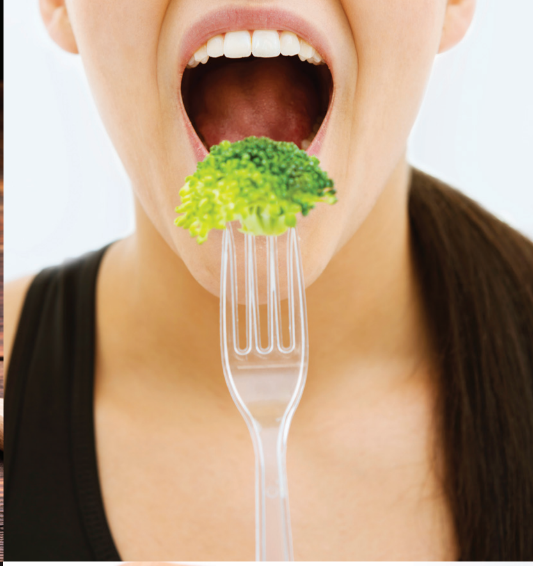
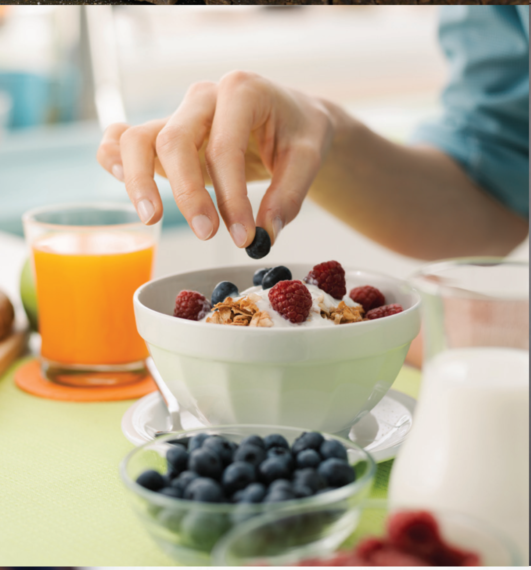
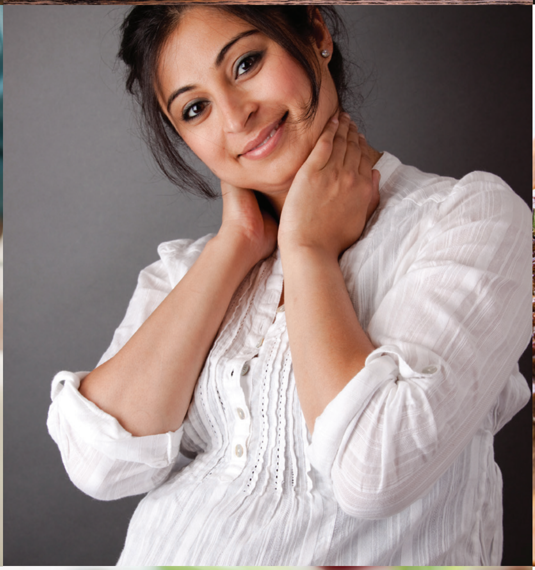
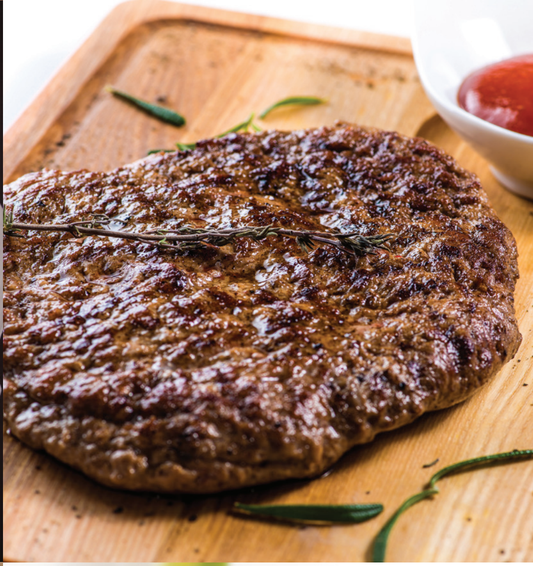
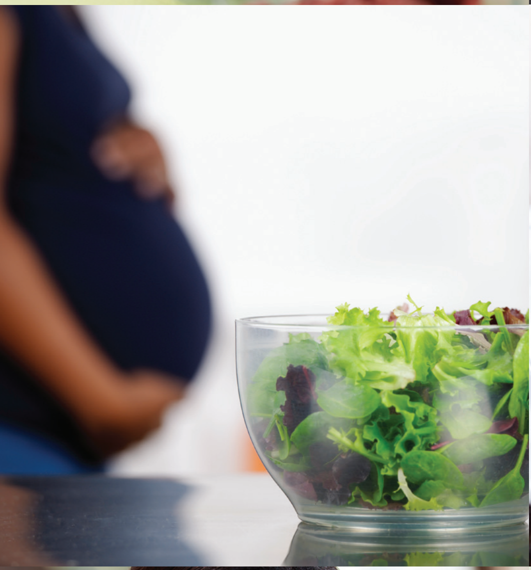
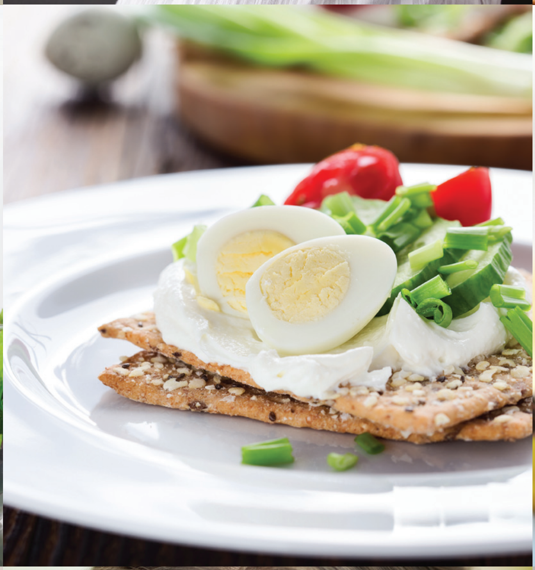
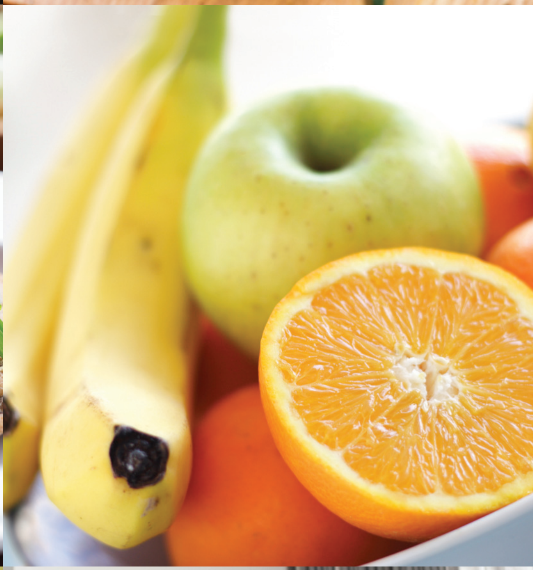
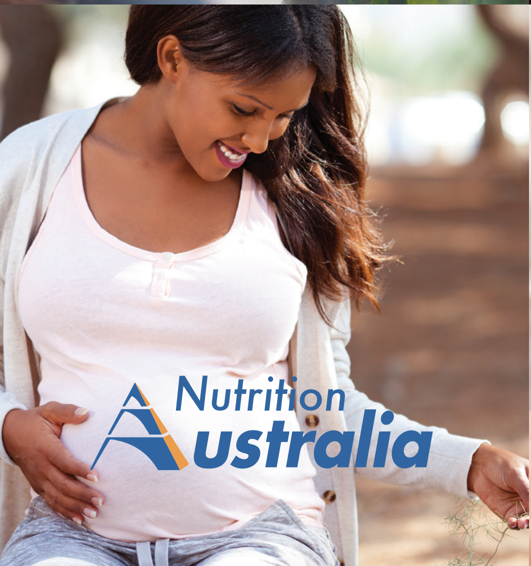
Nutrition Australia
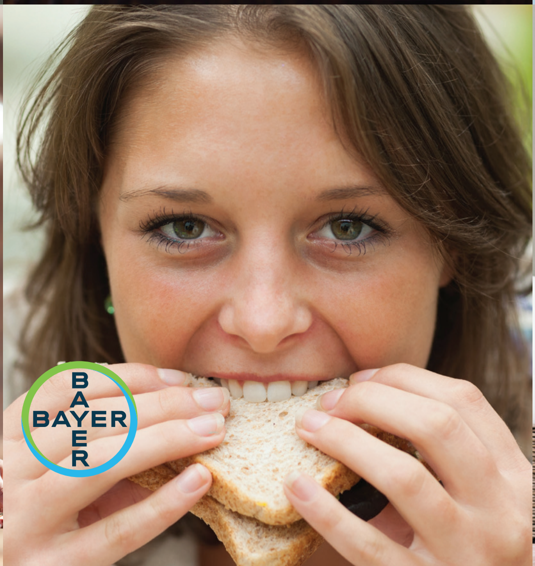
BAYER
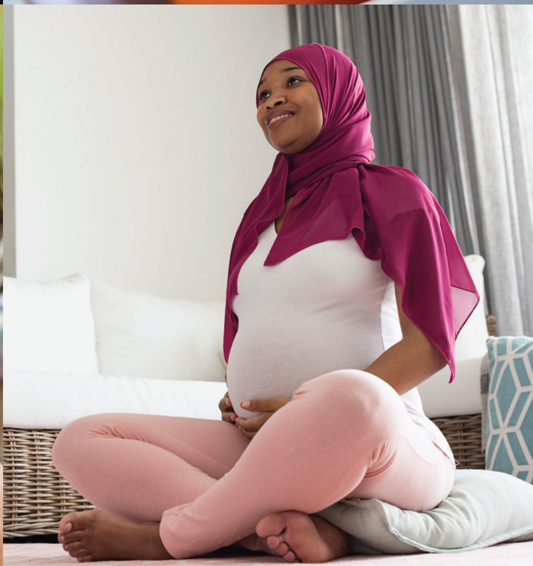

HEALTHY EATING WHEN YOU'RE PREGNANT

Now that you're pregnant it's the ideal time to review your diet. What you eat during pregnancy affects your own health and wellbeing, your developing baby and your baby's health later in life.

When pregnant you only need to increase how much you eat by a small amount, but you do need more of certain nutrients.

You will get most of the nutrients you need if you eat regular meals and include foods from the five food groups each day. Each food group provides different nutrients. The food groups are:

- vegetables
- fruit
- grains
- dairy foods and calcium fortified plant milks (such as soy milk)
- meats and meat alternatives (chicken, fish, eggs, tofu, legumes, nuts and seeds).

Most women will also need folic acid and iodine supplements as it is difficult to get enough of these from food alone.

See the table at the end of the fact sheet if you would like to know more about the recommended serves to eat from the food groups.

Tips on how to include a variety of foods in your everyday meals

Aiming for half plate of vegetables, a quarter of protein and quarter of carbohydrate can help you choose the right variety and balance of food.

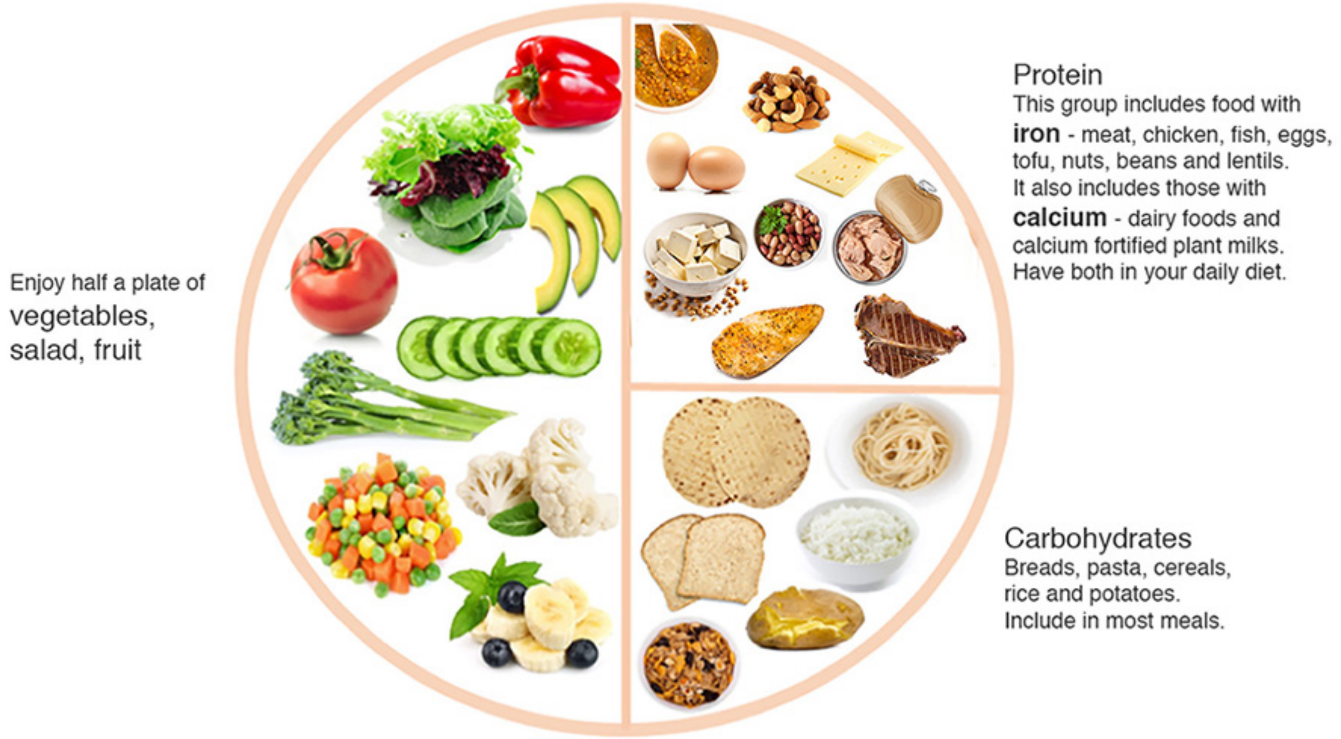

Below are examples of food to include in your daily meals, that is, a carbohydrate, a protein and fruit and vegetables. Choose foods from each column when preparing your breakfast, lunch and dinner.

Carbohydrate	+	Protein	+	Vegetables, salad, fruit
Grain- based foods and starchy vegetables		Dairy foods, eggs, nuts, nut butters, fish and seafood, legumes, chicken and meat		

Breakfast ideas

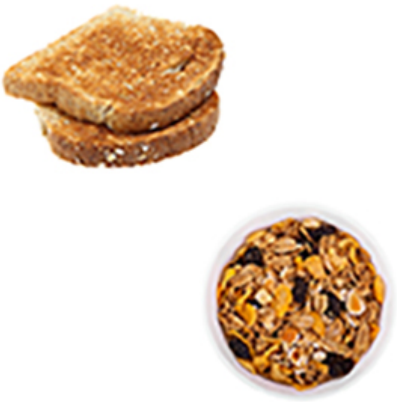

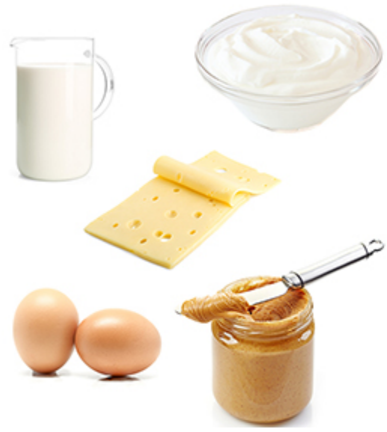

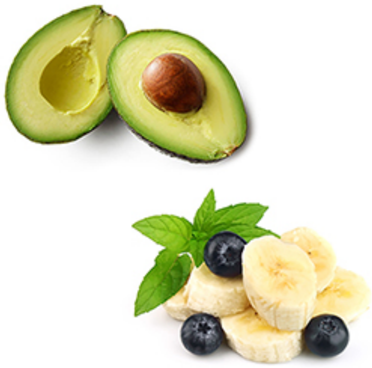

Lunch and dinner ideas

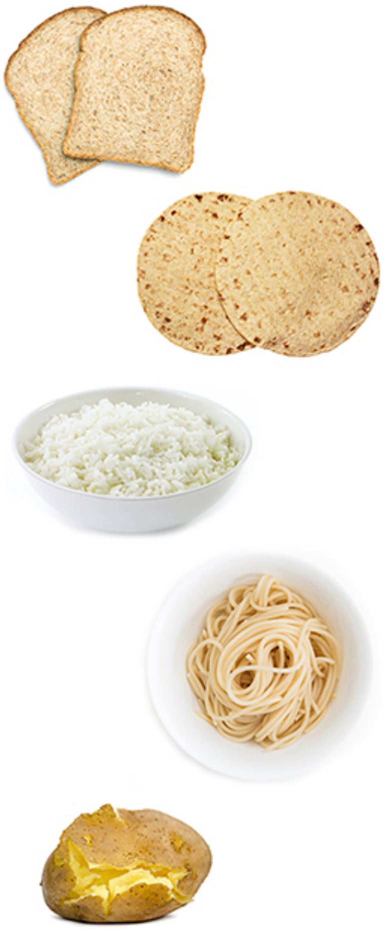

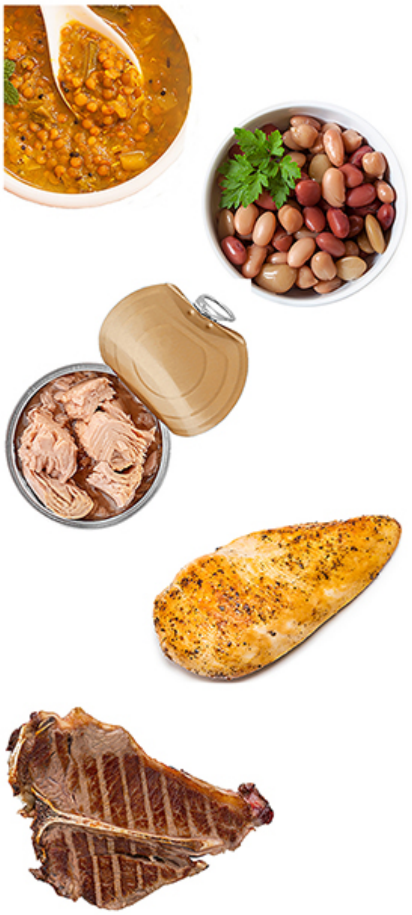

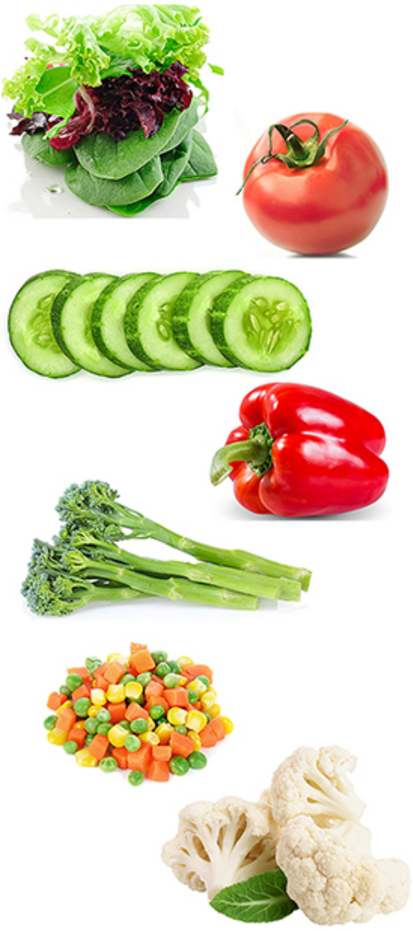

Below are nutritious snack ideas if you are hungry or are unable to eat enough at mealtimes.

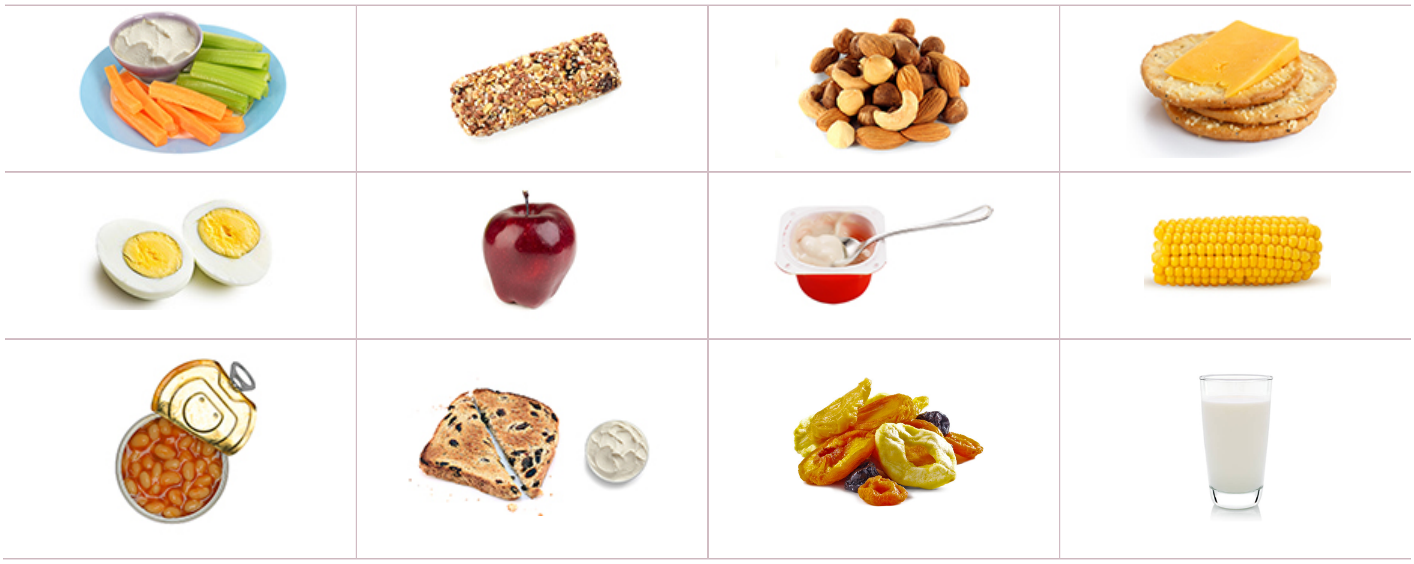

If nausea is affecting your food choices eat what you can manage and get back on track when you feel better. *See the fact sheet Nausea and vomiting in pregnancy (morning sickness) for more information.*

Important nutrients during pregnancy

Folic acid

Folic acid (also called folate) is a vitamin needed to build your baby's cells. In early pregnancy it can help reduce the risk of certain birth defects such as spina bifida.

It's difficult to get enough folate from food alone so you will need to take a supplement. Start taking folic acid when planning your pregnancy and continue for at least the first 3 months after you are pregnant. Take either a daily folic acid supplement (400 micrograms) or a pregnancy multivitamin. Some women with particular health conditions or who are overweight may be advised to take a higher dose of folic acid.

Folate is important throughout pregnancy. Green leafy vegetables, salad greens, fruit, wholegrain breads and cereals, fortified breakfast cereals, legumes and nuts are a good source of this important nutrient.

Iodine

Iodine is important for the developing brain and nervous system of your baby. As more is needed during pregnancy and breastfeeding, and it's hard to get enough from food, a daily supplement of 150 microgram of iodine is recommended. Most pregnancy multivitamins contain this dose.

Supplements are also available that contain both iodine and folic acid. **Do not** take kelp (seaweed) tablets as they may contain too much iodine. If you have a pre-existing thyroid condition speak to your doctor before taking an iodine supplement.

Protein

Protein helps build all body tissues. Aim to include protein-containing foods in each meal. Meat, chicken, fish, eggs, dairy foods, nuts and legumes (dried beans and lentils) and food made from them such as tofu are protein sources and will help your iron and calcium intake as well.

Iron

During pregnancy your body needs more iron to form red blood cells for yourself and your baby. Lack of iron can cause tiredness and if severe can increase the risk of premature birth and low birth weight babies. Meat, chicken and fish are good sources of iron. Legumes (lentils and dried beans), nuts, whole grains and green leafy vegetables also contain iron.

Iron from plant sources is not easily absorbed by the body. Eating vitamin C-rich foods at the same meal can improve this; foods such as citrus fruits, berries, tomatoes, capsicum.

If tests during pregnancy show that you are low in iron, you may need to take an iron supplement.

See the fact sheet Iron in Pregnancy for more information.

Calcium

Calcium helps form healthy bones. The richest source of calcium are dairy foods (note: low fat types have as much calcium as full fat versions). Fish with edible bones such as canned salmon and sardines, as well as firm tofu are also good sources. There are smaller amounts of calcium in other foods but most people get the majority of their calcium from dairy foods.

If you drink soy or other milks, such as almond or rice milk, check the label and choose a brand with the same amount of calcium as cow's milk (at least 100mg per 100ml). You need 2 to 3 serves of calcium-rich foods each day. If you don't eat dairy foods or other calcium-fortified milks, talk to you dietitian, midwife or doctor about whether you need calcium supplements.

Vitamin D

Vitamin D helps the body to absorb and use calcium. It works together with calcium to build your baby's bones. Only a small amount of vitamin D comes from food (found in oily fish, egg yolks, margarine, milk).

Sunlight helps your body make its own vitamin D. If you are indoors a lot, have darker skin or cover most of your body in clothing your vitamin D levels may be low. Very low levels can cause bone weakness and muscle pain in women and skeletal abnormalities (called rickets) in their babies. If you are at risk you may need to take a vitamin D supplement.

Multivitamin supplements

A balanced diet is the best way to meet the increased nutrient requirements of pregnancy but if you are unable to eat well, a pregnancy multivitamin supplement may help. A multivitamin supplement can also be a convenient way of getting enough folic acid and iodine if you are not already taking these supplements.

Check that any supplements you take are suitable for pregnancy. Multivitamin supplements not designed for pregnancy may contain too much vitamin A. Ask your pharmacist, doctor or dietitian for advice if you are unsure about supplements.

Omega-3 fats

Omega-3 fatty acids are needed for healthy brain, nerve and eye development in your baby and may have other health benefits. They are found in fish, especially oily fish like tuna, salmon, sardines and mackerel. Walnuts, chia seeds, linseeds (also called flax seeds) and soybeans also contain omega-3 fats.

Eating fish 2 to 3 times a week will help meet your growing baby's needs. Some types of fish may contain too much mercury and should not be eaten often.

- Limit shark (flake), marlin, broadbill or swordfish to no more than one serve per fortnight. Eat no other fish that fortnight.
- Limit orange roughy (deep sea perch) or catfish to one serve per week. Eat no other fish that week.
- Some pregnancy multivitamins include omega-3 fats. Omega-3 supplements are also available but avoid fish liver oils as they contain too much vitamin A (retinol).

If you're vegetarian or not feeling like meat

You can still meet your nutritional needs if you are vegetarian or avoiding animal foods due to nausea or taste changes. Just take care to include foods that contain enough protein, iron, calcium and vitamin B12.

Protein and iron

Food sources: nuts, seeds and legumes (dried beans and lentils) and food made from them such as tofu are sources of protein and iron.

Other iron-containing foods: wholegrain cereals, iron-enriched breakfast cereals, wholegrain breads and green leafy vegetables will help boost your iron intake.

Include vitamin C-rich fruits and vegetables in your meals to boost iron absorption.

Calcium

If you're not eating dairy foods use calcium-fortified soy or other plant milk or take a calcium supplement.

Vitamin B12

Vitamin B12 is needed for blood cell, nerve and brain development of your baby. It is naturally present only in foods of animal origin. Vegans and vegetarians who eat few dairy foods or eggs are at risk of deficiency, especially as pregnancy and breastfeeding rapidly use body stores. Breastfed babies of vegan mothers are particularly at risk of B12 deficiency.

Women at risk should have their level checked and may need to take a B12 supplement.

Although it is added to certain brands of soy milk and meat substitutes the amount in these foods may not be enough if few other sources of B12 are eaten.

If you are concerned please talk to your dietitian or doctor.

See also the fact sheet *Healthy eating when you're pregnant: Information for vegetarians and vegans*.

Foods to avoid or limit

As well as limiting fish high in mercury, pregnant women should also be aware of the following food safety issues.

Food-borne infections

Pregnant women are at greater risk of some types of food poisoning and infections that can be passed on to the baby. To reduce your risk:

- Use good food handling practices such as using separate chopping boards for raw and cooked foods.
- Don't leave leftover cooked food on the bench for too long. Place it in the fridge once it has stopped steaming.
- Avoid foods that may contain the listeria bacteria such as sandwich meats, soft cheeses (brie, camembert, ricotta, feta and blue cheese), soft serve ice-cream, pate, pre-prepared salads, smoked salmon, uncooked seafood and pre-cooked prawns. Freshly cooked seafood is safe. Listeria is killed by cooking food to boiling point, so when cooking or reheating foods, make sure they are steaming hot.
- Raw meat and cat poo can carry toxoplasmosis. To reduce your risk of infection, thoroughly cook meat, wear rubber gloves if handling cat litter and wash hands after gardening or handling pets.
- Avoid undercooked eggs and meat to limit risk of salmonella food poisoning, which in rare cases can affect the baby. Cook eggs until yolk and white are firm.
- Sesame seeds are also a salmonella risk so avoid eating sesame seeds and ready to eat products such as tahini, halva and hummus. Sesame seeds that have been heat treated are safe to eat.

Alcohol

Not drinking alcohol is the safest option.

Caffeine

Tea and coffee contain caffeine. These are safe to drink in moderation, for example, 1 to 2 coffees or up to 5 cups of tea per day. Energy drinks can contain large amounts of caffeine or guarana (a source of caffeine) so should be limited.

Liver and vitamin A

Limit liver to 50g a week as it can contain more than the recommended levels of vitamin A (retinol) for pregnant women.

See the fact sheet *Food Safety during Pregnancy* for more information.

How much weight should I gain?

Your recommended weight gain depends on your pre-pregnancy body mass index (BMI). To calculate your BMI divide weight (in kilos) by height (in metres) squared. Check the table below for the recommended weight gain for your BMI range.

If you find you have gained a lot of weight early in pregnancy, aim to slow your weight gain down to the recommended monthly gain.

Dieting is not recommended, instead limit intake of high fat and high sugar foods and do some daily exercise such as walking. Speak to your doctor, midwife or physiotherapist if you are unsure what type of exercise is appropriate for you.

See *Weight and pregnancy* fact sheet for more information and tips on what to eat if you are worried you are gaining too much or too little weight.

Pre-pregnancy	BMI European	BMI Asian	Recommended weight gain during pregnancy	Weight gain per month in 2nd and 3rd trimesters*
Underweight	Less than 18.5		12.5 to 18kg	2 to 2.5kg
Normal weight	18.5–24	18.5 to 22.9	11.5 to 16.0kg	1.5 to 2.2kg
Overweight	25–29	23 to 27.5	7 to 11.5kg	1 to 1.4kg
Obese	30 and above	Above 27.5	5 to 9kg	0.7 to 1.2kg

*The average weight gain in the first three months is 0.5 to 2kg.

Where to get more information

If you have questions about what to eat or weight gain during pregnancy ask for a referral to a dietitian.

The Women's website
For more information on nutrition and pregnancy visit
www.thewomens.org.au/hi-healthy-pregnancy

The following fact sheets are also available for download:

- Healthy eating when you're pregnant with twins
- Iron in pregnancy
- Nausea and vomiting in pregnancy (morning sickness)
- Coping with common discomforts of pregnancy
- Healthy eating when you're pregnant: Information for vegetarians and vegans
- Weight gain during pregnancy

Food Standards Australia New Zealand
This website has useful information for consumers. Search for 'Fish and mercury', 'Listeria and food' and 'Food poisoning'
www.foodstandards.gov.au/consumer/

Eat for Health
Visit the Australian Dietary Guidelines website for advice and resources about healthy eating.
www.eatforhealth.gov.au

Healthy foods guide

Use the following table as a guide to foods you should include in your daily diet.

Note: this information is taken from the Australian Dietary Guidelines

Food group	Daily serves	Each item is an example of a serve
Vegetables & Legumes	5	½ cup vegetables<br>1 cup salad<br>½ cup legumes (cooked)
Fruit	2	1 medium or 2 small pieces fruit<br>1 cup tinned fruit or fruit salad<br>1 tablespoon dried fruit
Meat and<br>meat alternatives	3 ½	65g cooked meat<br>80g cooked chicken<br>100g cooked fish fillet of 1 small can of fish<br>2 eggs<br>1 cup legumes canned or cooked legumes/beans - lentils, chickpeas<br>170g tofu<br>1 tablespoon (30g) nuts
Dairy or alternatives such as soy or almond milk	2 ½	1 cup milk<br>Small tub (¾ cup) yoghurt<br>2 slices (40g) cheese
Grain foods (breads & cereals)	8 ½	1 slice bread, ½ bread roll, 1 chapatti,<br>½ Lebanese bread, 3 crisp breads<br>1 cup cereal, ½ cup porridge (cooked)<br>½ cup rice, pasta, noodles (cooked)
Unsaturated oils and spreads	Optional	1 to 2 tablespoons per day

Limit foods that are high in added fats and sugars, such as cakes, biscuits, pastries, high fat takeaway foods and sugar-sweetened soft drinks and juices. While these are fine in small amounts, in large amounts they can cause excess weight gain or take the place of more nutritious foods.

DISCLAIMER This fact sheet provides general information only. For specific advice about your baby or your healthcare needs, you should seek advice from your health professional. The Royal Women's Hospital does not accept any responsibility for loss or damage arising from your reliance on this fact sheet instead of seeing a health professional. If you or your baby require urgent medical attention, please contact your nearest emergency department. © The Royal Women's Hospital 2019–2021
