# Supplementary material for: Co‐Designing a Culturally Tailored Nutrition Resource With African Migrant Women and Healthcare Professionals in Australia
Source: Health Expect. 2026 Mar 25;29(2):e70649. doi: 10.1111/hex.70649 (PMC13087440; doi:10.1111/hex.70649)
Supplement: Supplementary file 2 — Supporting file 2: Workshop guide. [file HEX-29-e70649-s001.docx]

**Workshop guide**

**Codesign workshop: Designing culturally acceptable pregnancy nutrition education resources with African migrant women**

# Workshop duration: 120 minutes

1. **Welcome and introduction (5 minutes)**

Overview of the purpose of the workshop: This workshop aims to gather your input on pregnancy nutrition resources and collaborate on designing resources that are culturally relevant and acceptable to African migrant women.

Brief introductions from facilitators and participants.

*Facilitator play the pre-workshop video*

# Activity 1: Warm-up (10 minutes)

Share a piece of nutrition advice that you would give to a friend who is expecting her first baby. This can be something useful you have experienced or heard from others.

*Now that we have shared personal advice, let’s focus on pregnancy nutrition resources.*

# Activity 2: Review of existing resources (30 minutes)

Participants will move into breakout rooms to review the resources provided by the facilitators

# Discussion points:

- - Have you used any of these resources before? (If yes, what was your experience? If not, why?)
  - What do you like about these resources?
  - What do you dislike about these resources?
  - What do you see as gaps in the information provided?
  - How could these resources be improved to better suit your cultural needs during pregnancy?

*Discuss as a group, feel free to write down your thoughts and nominate someone to present what you have written.*

***Facilitators’ role:*** *Ensure that all participants are actively engaging in the discussion.*

***Group discussion feedback (15 minutes)***

*Since you have provided detailed review and information on the existing resources, kindly engage in the reflection below.*

# Activity 3: Individual reflection on resource preferences (10 minutes)

- - What type of resource (s) would best suit your needs during pregnancy?
  - How could such resources be made more culturally accepted?

*Share your response with facilitators.*

# Activity 4: Codesigning a pregnancy nutrition resource (30 minutes)

Participants will move into breakout rooms to collaboratively design a pregnancy nutrition resource that would be culturally suitable for African migrant women. Draw inspiration from the resources you reviewed earlier, and the responses provided in Activity 2.

***Note:*** *This is a creative exercise, don’t worry about making it perfect; the goal is to generate ideas. Consider factors like literacy levels, language, and accessibility as you design.*

# Activity 5: Group presentation (15 minutes)

Open discussion on the resource designed by the participants.

***Facilitators’ role:*** *Encourage discussion on whether participants agree or disagree with what is been presented and invite additional contributions or suggestions.*

# Closing and summary (5 minutes)

- - Summarise the key takeaways from the workshop
  - Invite final comments from participants.
  - Provide information about the next workshop, which will involve collaboration with healthcare professionals.

**Thank you for attending.**

**Workshop guide**

**Codesign workshop: Designing culturally acceptable pregnancy nutrition education resources with healthcare professionals**

# Workshop duration: 90 minutes

1. **Welcome and introduction (5 minutes)**

Overview of the purpose of the workshop: This workshop aims to gather your expert opinions and skills in designing culturally acceptable pregnancy nutrition education resources for African migrant women.

Brief introductions from facilitators and participants

*Facilitator play the pre-workshop video*

# Activity 1: Warm-up (10 minutes)

This is a reflection activity. Reflect on your interactions with African migrant women during pregnancy care. Have your services, particularly those related to nutrition, been

received as culturally relevant? Why or why not?

*Now that we have considered pregnancy nutrition education, let’s turn our attention to the specific pregnancy nutrition resources available to these women.*

# Activity 2: Pregnancy nutrition education resources (10 minutes)

List the pregnancy nutrition education resources you use or have available when providing advice to African migrant women.

- - What comments have you heard from women about the appropriateness of these resources?
  - What do you perceive as barriers to using these resources for women of different cultural backgrounds in your care?

***Facilitators’ role:*** *Share examples of existing pregnancy nutrition education resources with participants.*

# Activity 3: Review of existing resources (30 minutes)

Participants will share their views with the facilitator about the existing resources. The questions below will be used to prompt discussion.

# Discussion points:

- - Are these resources culturally tailored to meet the needs of pregnant African migrant women? Why or why not?
  - How could these resources be culturally tailored to better meet their needs?
  - What type of resource would be useful to you when delivering nutrition advice to African migrant women?

*After reviewing the existing resources, the next step will be to co-design a pregnancy nutrition education resource for African migrant women.*

# Activity 4: Codesigning a pregnancy nutrition resource (30 minutes)

Participants will design a pregnancy nutrition resource that would be culturally suitable for African migrant women. Draw inspiration from the resources you reviewed earlier, and the personal reflections in Activity 1.

***Note:*** *This is a creative exercise, don’t worry about making it perfect; the goal is to generate ideas. Consider factors like literacy levels, language, visual elements and accessibility as you design.*

# Closing and summary (5 minutes)

- - Summarise the key takeaways from the workshop
  - Invite final comments from participants.
  - **Ask:** What is one thing you will take back to your workplace?
  - Provide information about the next workshop, which will involve collaboration with African migrant women.

**Thank you for attending.**

**Workshop guide**

**Joint workshop**

# Workshop duration: 120 minutes

1. **Welcome and introductions**

Provide an overview of the purpose of the workshop

1. **Activity 1: Warm-up activities with both groups**

- **For African migrant women:** What is one food or meal you enjoyed during pregnancy or postpartum? What made it special or important to you?
- **For Healthcare professionals:** What is one food or meal you have suggested or recommended to African migrant women during pregnancy or postpartum, and why do you recommend it?

# Activity 2: Overview of findings from workshops with African migrant women and healthcare professionals

- - Present the findings from workshops conducted with both African migrant women and healthcare professionals.

***Facilitators’ role:*** *Ask participants to confirm the accuracy of the findings presented and invite their comments and questions.*

# Activity 3: Deliberation on the co-designed resources

- - Participants will move into breakout rooms to deliberate and finalise, as a team, the potential culturally tailored pregnancy nutrition resource, including its content and format.

***Group discussion feedback***

# Activity 4: Open discussions with participants

- - Discuss the next steps for action
  - Are there any suggestions for future research?
  - Any final comments?

# Closing remarks

Thank you for attending. Are you interested in the workshop transcripts? Individual gift voucher card will be sent after the workshop.

**Thank you once again.**
