# Supplementary material for: Co‐Designing a Culturally Tailored Nutrition Resource With African Migrant Women and Healthcare Professionals in Australia
Source: Health Expect. 2026 Mar 25;29(2):e70649. doi: 10.1111/hex.70649 (PMC13087440; doi:10.1111/hex.70649)
Supplement: Supplementary file 3 — Supporting file 3: Coding tree showing the analytic pathway. [file HEX-29-e70649-s003.docx]

**Supplementary file 3: Coding tree showing the analytic pathway**

| **Codes** | **Categories** | **Theme** | **Sample Quotes** |
| --- | --- | --- | --- |
| Lack of food descriptions, limited translations, overly wordy, too generalised, absence of African/familiar food information, unsure of alternatives to familiar foods, targeted to Australian audience, unable to meet pregnancy nutritional needs | Lack of cultural relevance, resources too Western-focused, absence of African foods | Theme 1: Cultural misalignment in existing nutrition resources | *“…the kinds of foods that we eat is a bit different from the kinds of foods included in these flyers…”* (Woman 7)  *“…they're not speaking directly to what we consider as a meal in Africa”* (HCP 4) |
| Inclusion of African foods in Australia, food quantities in relatable measurements, address taboos and myths, suitable substitutes, linguistic accessibility, use of visuals and images, where to purchase African foods, explain nutritional benefits | Culturally tailored content, accessible communication and presentation | Theme 2: Translating pregnancy nutrition guidance into culturally relevant resources | *“…foods that are healthy and that we are familiar with… pictures will work…”* (Woman 4)  *“…using clear language, simple terms and no jargon…”* (HCP 4) |
| Country-specific sections, FAQ on nutrition, food substitutions and regional variation, portion and calorie guidance, demystifying taboos, visual balanced plate, recipes and preparation steps, tool for HCP, direct information, digital and language translation, conversation section | Digital accessibility, flexible access, practical adaptation, inclusivity, clinical usability, actionable content, practical teaching tool | Theme 3: Designing practical and usable pregnancy nutrition resources | *“…having resources and better insight into what they're actually eating, we need it”* (HCP 5)  *“…this pound of meat or this kilo of meat…this kilo in a week, do not have more than this because these are the calories you get”* (Woman 3) |
