# Supplementary material for: Co‐Designing a Culturally Tailored Nutrition Resource With African Migrant Women and Healthcare Professionals in Australia
Source: Health Expect. 2026 Mar 25;29(2):e70649. doi: 10.1111/hex.70649 (PMC13087440; doi:10.1111/hex.70649)
Supplement: Supplementary file 4 — Supporting file 4: Culturally tailored features with accompanying participant quotes. [file HEX-29-e70649-s004.docx]

**Supplementary file 4: Culturally tailored features with accompanying participant quotes**

| **Feature category** | **Specific features described by participants** | **Participant quotes** |
| --- | --- | --- |
| Content features | **African foods with their alternatives** | *‘With the foods listed, you could say like if there's foods that aren't accessible here, like what an alternative to that is as well that is easily accessible in Australia’* [Website]  *‘…short little recipe list of how to prepare the Western food alternatives so people understand how to use them’* [Pamphlet] |
|  | **Calorie and weight tracking** | *‘So the app should be able to help us in a way to watch our weight to be able to know the amount of calories we are taking per day’* [Mobile application] |
|  | **Country-specific and general nutrition information** | *‘African is quite big and even in a country we have hundreds of tribes and all that. So in order to make it easier, I will say that if there is an app, if possible we should be able to click on countries. Maybe there should be a section that will say what country in African are you from?’* [Mobile application] |
|  | **Food recipes with their nutritional values** | *‘…things about the food should be listed there maybe the nutritional value you know, maybe how to prepare it…like the recipe for that can be there’* [Mobile application] |
|  | **Inclusion of regional foods** | *‘When we are talking about African migrant in their different countries…the website. It should be able to contain… foods from different regions of Africa so that if I am logging on to the website I will be able to maybe see something I can relate to as someone from where I am from…* *not an extensive list of things, but some examples from each region of what is a balanced meal?’* [Website] |
|  | **Information on exercise** | *‘…there should be a section for exercises…this is general for all Africans. So this is like exercises. You should know the kind of exercises that's good for pregnant women’ [Mobile application]* |
|  | **Location to purchase African foods** | *‘I think information of where to buy the foods as well is quite important because it's important that whatever is recommended is accessible’* [Website] |
|  | **Pictorial representation of a balanced meal** | *‘…if we included like a plate and it said like what a balanced plate of food looks like…it has like the grain section and it has a list of all of the grains that you could have and then people could pick and choose what's actually relevant to them’ [Website]*  *‘Picture of a plate with proportions for a balanced meal…featuring a traditional meal or traditional foods, but with a proportion and guide of protein, carbohydrates and fats to say what does a balanced meal look like?’* [Pamphlet] |
|  | **Portions and calorie recommendations** | *‘…we spoke about calories too, maybe there should be a section that will tell us, for example, this pound of meat or this kilo of meat. It's good for you to have at least this kilo in a week, do not have more than this because these are the calories you get’* [Mobile application]  *‘…it should only contain what should be consumed in what proportion and the food classes… So the food guide itself would not even be vague at all. It has to be very direct and this is what you should be consuming in pregnancy’* [Food guide] |
| Design and language features | **Plain English with technology-assisted translations** | *‘In plain English would be very, very easy for most of the women to navigate’* [Website]  *‘I think there should also be a function where you can like with AI read it to you so if like reading was an issue as a challenge as well then that could be an option as well to make it more inclusive’* [Mobile application] |
|  | **Visually appealing, clear and concise** | *‘…making it more clear like ensuring that the information is very clear in bullet points, not like essays…so that it can capture people's attention with ease’* [Mobile application]  *‘The most important things were visually appealing and minimal writing’* [Pamphlet] |
